# Acyl-group specificity of AHL synthases involved in quorum-sensing in *Roseobacter* group bacteria

**DOI:** 10.3762/bjoc.14.112

**Published:** 2018-06-05

**Authors:** Lisa Ziesche, Jan Rinkel, Jeroen S Dickschat, Stefan Schulz

**Affiliations:** 1 Institute of Organic Chemistry, Technische Universität Braunschweig, Hagenring 30, 38106 Braunschweig, Germany; 2 Kekulé-Institute of Organic Chemistry and Biochemistry, University of Bonn, Gerhard-Domagk-Str. 1, 53121 Bonn, Germany

**Keywords:** *Dinoroseobacter shibae*, fatty acid composition, *N*-acylhomoserine lactones, quorum sensing, *Phaeobacter inhibens*

## Abstract

*N*-Acylhomoserine lactones (AHLs) are important bacterial messengers, mediating different bacterial traits by quorum sensing in a cell-density dependent manner. AHLs are also produced by many bacteria of the marine *Roseobacter* group, which constitutes a large group within the marine microbiome. Often, specific mixtures of AHLs differing in chain length and oxidation status are produced by bacteria, but how the biosynthetic enzymes, LuxI homologs, are selecting the correct acyl precursors is largely unknown. We have analyzed the AHL production in *Dinoroseobacter shibae* and three *Phaeobacter inhibens* strains, revealing strain-specific mixtures. Although large differences were present between the species, the fatty acid profiles, the pool for the acyl precursors for AHL biosynthesis, were very similar. To test the acyl-chain selectivity, the three enzymes LuxI_1_ and LuxI_2_ from *D. shibae* DFL-12 as well as PgaI_2_ from *P. inhibens* DSM 17395 were heterologously expressed in *E. coli* and the enzymes isolated for in vitro incubation experiments. The enzymes readily accepted shortened acyl coenzyme A analogs, *N*-pantothenoylcysteamine thioesters of fatty acids (PCEs). Fifteen PCEs were synthesized, varying in chain length from C_4_ to C_20_, the degree of unsaturation and also including unusual acid esters, e.g., 2*E*,11*Z*-C18:2-PCE. The latter served as a precursor of the major AHL of *D. shibae* DFL-12 LuxI_1_, 2*E*,11*Z*-C18:2-homoserine lactone (HSL). Incubation experiments revealed that PgaI_2_ accepts all substrates except C_4_ and C_20_-PCE. Competition experiments demonstrated a preference of this enzyme for C_10_ and C_12_ PCEs. In contrast, the LuxI enzymes of *D. shibae* are more selective. While 2*E*,11*Z*-C18:2-PCE is preferentially accepted by LuxI_1_, all other PCEs were not, except for the shorter, saturated C_10_–C_14_-PCEs. The AHL synthase LuxI_2_ accepted only C_14_ PCE and 3-hydroxydecanoyl-PCE. In summary, chain-length selectivity in AHLs can vary between different AHL enzymes. Both, a broad substrate acceptance and tuned specificity occur in the investigated enzymes.

## Introduction

The *Roseobacter* group, a subgroup of the *Rhodobacteraceae* family, constitutes an important class of Gram-negative marine bacteria, occurring in many different habitats [1–2], in fresh water as well as on surfaces [3]. They can produce a variety of secondary metabolites, including antibiotics [4–5], volatile compounds [6–7], oligohydroxybutyrates [8] and a range of *N*-acylhomoserine lactones (AHLs) [8–10]. AHLs are quorum-sensing signaling compounds that are used for cell–cell communication to regulate several physiological traits regulated by cell density, the ‘quorum’ [11–16], in roseobacters, e.g., in the production of the antibiotic tropodithietic acid in *Phaeobacter inhibens* [15] and cell differentiation in *Dinoroseobacter shibae* [14]. *Roseobacter* group AHLs are characterized by saturated, unsaturated and sometimes oxygenated acyl chains ranging in length between C_8_ and C_18_ [8] with the exception of the aromatic *p*-coumaroylhomoserine lactone produced by *Rugeria pomeroyi* DSS-3 [17].

In a recent analysis we showed the AHL presence in 19 out of 24 *Roseobacter* group bacterial strains isolated from macroalgal surfaces [8]. The most widespread AHL was 7-tetradecenoylhomoserine lactone (7-C14:1-HSL), present in seven strains. No clear correlation between phylogeny and AHL occurrence was observed. In some strains only one AHL was detected, while others such as *P. gallaeciensis* BS107 produced eight different AHLs [8].

**Scheme 1:**
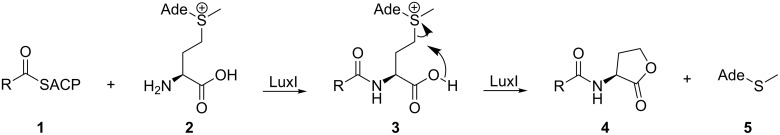
Biosynthesis of AHLs by ACP-dependent LuxI type enzymes.

The biosynthesis of AHLs is mediated by the enzyme LuxI or its homologs, and often accompanied by a regulator protein, LuxR [18–19]. An ACP-bound fatty acid acyl group **1** is transferred onto the amino group of *S*-adenosylmethionine (SAM, **2**) that is followed by substitution of the good leaving group 5’-deoxy-5’-thiomethyladenosine (**5**) of the thioester group, leading to homoserine lactone **4** formation (Scheme 1). Recently a LuxI-homolog, BjaI [20] preferring acyl-coenzyme A (CoA) substrates instead of the common ACP precursors, was characterized [21].

The LuxI-type enzymes are the most widespread and best understood AHL synthases. Four structures of LuxI-type enzymes have been published, covering both ACP and CoA-dependent structures with various chain lengths and different oxidation states of the acyl chain at C-3 [21–24]. A great diversity among AHL synthases is observed. The preference for unsubstituted, 3-oxo or 3-hydroxyacyl precursors is mediated by binding interactions inside the active site of AHL synthases [18,21]. Investigations on the chain-length selectivity of the AHL synthases are limited. BjaI can accept substrates ranging from isovaleryl-CoA, the native substrate, up to isononanoyl-CoA [21].

Three different LuxI homologs, LuxI_1_, LuxI_2_, and LuxI_3_, occur in *Dinoroseobacter shibae* DFL-12 [14]. Recently, we were surprised to find that the structures of AHLs synthesized by a LuxI homolog from *D. shibae* DFL-12 depended on the host in which the enzyme was expressed [10]. Expression of LuxI_1_ in *E. coli* led to a predominant formation of a 2:1:0.3 mixture of 9-C18:1-homoserine lactone (HSL), C16:0-HSL and C14:0-HSL, while the overexpression in its parent strain furnished the native product, 2*E*,11*Z*-C18:2-HSL, accompanied by 4% each of 9-C18:1-HSL and 2,9-C16:2-HSL. While the native substrates of LuxI_2_ and LuxI_3_ were not detected because of their low concentration, their overexpression in *E. coli* led to the production of a 6:1 mixture of 7*Z*-C14:1-HSL and C14:0-HSL for LuxI_2_ and no AHL formation for LuxI_3_ [10]. The differences between the AHLs in terms of chain length and degree of unsaturation prompted us to investigate the acyl-chain selectivity of LuxI-type enzymes in roseobacters. Does the enzyme have an inherent selectivity for a specific acyl-chain precursor or does it react unselectively with every acyl-precursor available? In the latter case the presence of the acyl precursors would determine the structure of the final AHL. To answer this question, the fatty acid composition of the native roseobacters was determined and compared to the AHLs produced. In addition, LuxI-type enzymes were heterologously expressed in *E. coli* and the purified recombinant enzymes were tested with different precursors to probe their selectivity. Both model organisms of the *Roseobacter* group, *P. inhibens* (formerly *P. gallaeciensis* [25]) DSM17395 and *Dinoroseobacter shibae* DFL-12, were investigated, together with closely related *P. inhibens* strains T5 and 2.10 [26], to investigate strain variability. Previously, the LuxI homolog PgaI_1_ from *P. inhibens* DSM 17395 has been characterized, producing *R*-3-OH-C10:0-HSL [15,27]. This strain produced additionally long chain AHLs such as C18:1-HSL [9] and contains a second AHL synthase, PgaI_2_ [28], probably involved in the biosynthesis of the long chain AHLs. Here we report on the characterization of PgaI_2_ from *P. inhibens* and of LuxI_1_ and LuxI_2_ from *D. shibae* by in vitro incubation experiments.

## Results and Discussion

The AHL production of four *Roseobacter* group strains was analyzed by a GC/MS-based method using XAD-16 as adsorbent in marine broth, developed by us [8]. The bacteria were isolated from different habitats: *D. shibae* DFL-12 was isolated from the dinoflagellate *Prorocentrum lima* [29], *P. inhibens* T5 was collected from a water sample of the German Wadden Sea [30], *P. inhibens* DSM17395 was isolated from seawater of larval cultures of the scallop *Pecten maximus* in Spain [25] and *P. inhibens* 2.10 stemmed from the surface of the green macroalga *Ulva australis* in Australia [31].

The results showed that *P. inhibens* 2.10 and *P. inhibens* DSM17395 produce the same four AHLs, 3-OH-C10:0-HSL as major components and known from previous analyses of *P. inhibens* [9,32], C16:0-HSL, C16:1-HSL, and C18:1-HSL (Table 1). *P. inhibens* T5 additionally produced 3-oxo-C10:0-HSL and C12:2-HSL with unknown location of the double bonds. *D. shibae* DFL-12 released C14:1-HSL, 3-oxo-C14-HSL, C18:1-HSL, and C18:2-HSL, similar to previous results [10,16].

**Table 1:** Presence of different AHLs in four strains of *Roseobacter* group bacteria.^a^

strain	3-OH- C10:0-HSL	3-oxo- C10:0-HSL	C12:2- HSL	C14:1- HSL	3-oxo- C14:0-HSL	C16:0- HSL	C16:1- HSL	C18:1- HSL	C18:2- HSL

*P. inhibens* 2.10	40.5					15.9	12.9	30.6	
*P. inhibens* DSM17395	87.1					3.9	2.8	6.2	
*P. inhibens* T5	37.3	5.0	16.2			4.9	10.0	26.6	
*D. shibae* DFL-12				5.8	13.6			9.3	71.3

^a^Relative amounts of AHLs for each strain in %.

**Figure 1:**
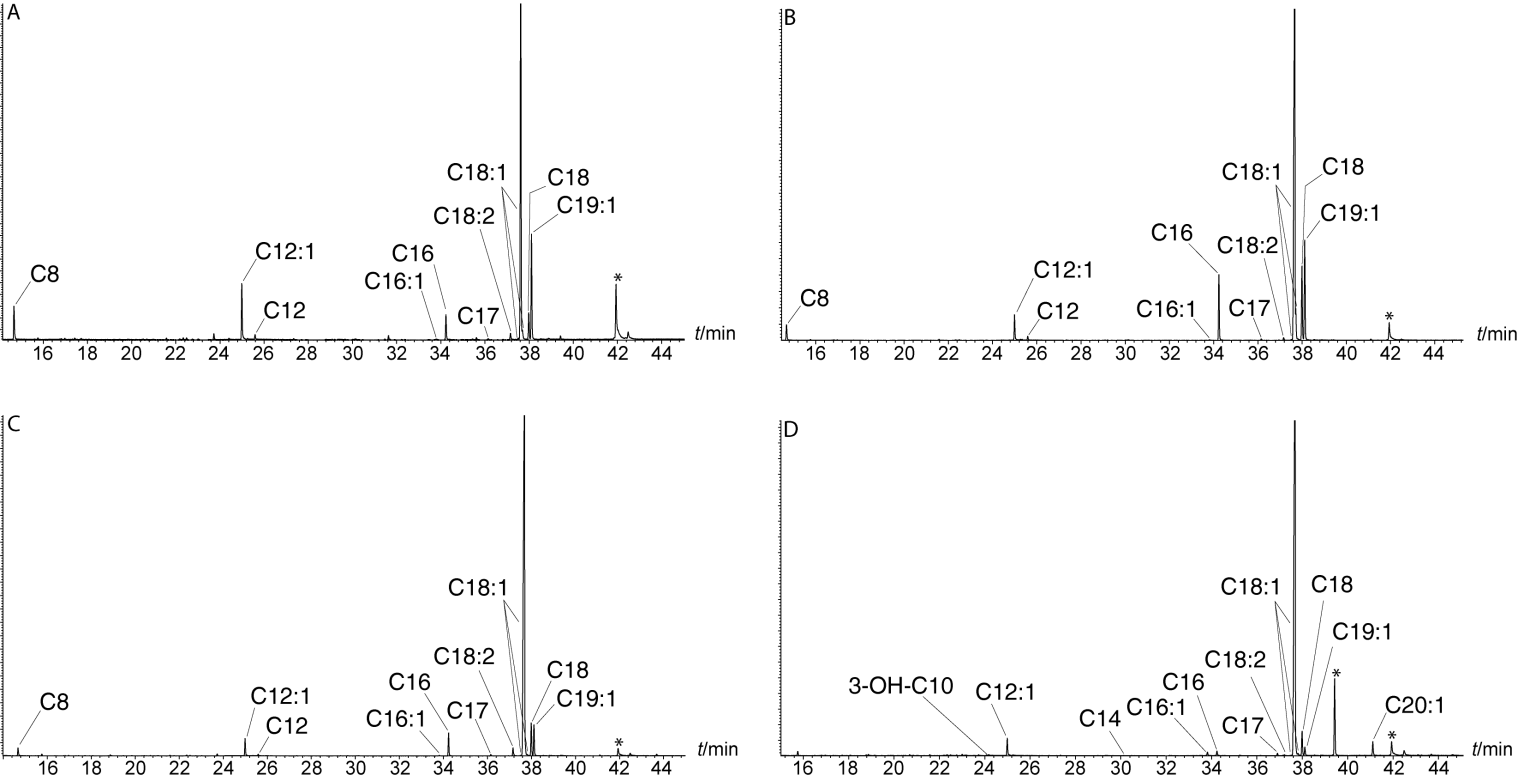
Total ion chromatograms of the FAME extracts of A) *P. inhibens* 2.10, B) *P. inhibens* DSM17395, C) *P. inhibens* T5 and D) *D. shibae* DFL-12. *other compounds.

In addition, the fatty acid profile of the four strains was determined. Therefore, bacterial colonies from agar plates were added to 20 μL of methanolic trimethylsulfonium hydroxide (TMSH) solution. This procedure lyses the bacteria and concomitantly transfers any bound or free fatty acid into its methyl ester (FAME) [33]. The extracts were analyzed by GC/MS (Figure 1). Short and long FAMEs were detected, ranging from methyl octanoate to methyl icosanoate (Table 2). The three *Phaeobacter* strains produced identical fatty acids. We identified FAMEs with a C8:0, C12:0, C12:1, C16:0, C16:1, C17:0, C18:2, C18:0, C18:1, and C19:1 chain, the three last ones being the most abundant. *D. shibae* DFL-12 showed a similar fatty acid production, but no FAMEs with C_8_ or C_12_ chains were detected. Instead, 3-OH-C10:0-HSL, C14:0, and C20:0 FAMEs occurred in addition.

**Table 2:** Presence of bound or free fatty acids in four different *Roseobacter* group strains detected as methyl esters.^a^

	*P. inhibens*			*D. shibae*	*P. inhibens*			*D. shibae*

	agar plate				liquid culture			

	2.10	DSM17395	T5	DFL-12	2.10	DSM17395	T5	DFL 12

C8:0	5.0	1.8	1.0		0.1	0.9	0.2	
3-OH-C10:0-HSL				0.1				0.5
C12:0	0.6	0.4	0.2		0.1	0.2	0.1	
5*Z*-C12:1	8.2	2.9	2.2	2.3	0.5	2.2	0.9	0.8
C14:0				<0.1				0.1
C16:0	3.8	7.5	3.0	0.6	4.3	2.6	2.2	0.3
9*Z*-C16:1	0.1	<0.1	<0.1	0.5	0.1	0.1	<0.1	0.2
C17:0	0.4	0.2	0.2	<0.1	0.1	0.1	0.1	<0.1
C18:0	3.9	8.6	4.6	2.6	2.1	1.8	1.8	3.4
11*Z*-C18:1	60.4	66.7	83.1	91.9	61.0	67.8	67.3	92.4
11*E*-C18:1	0.3	0.1	0.1	<0.1	0.1	0.1	0.1	0.1
13Z-C18:1	0.4	<0.1	<0.1	0.1	1.2	1.0	1.0	0.3
2*E*,11*Z*-C18:2	1.2	0.3	1.2	<0.1	3.2	1.8	2.9	<0.1
11Me-12*E*-C19:1	15.7	11.4	4.3	0.6	27.2	21.5	23.4	1.0
13*Z*-C20:1				1.2				0.9

^a^Relative peak areas of FAMEs for each strain in %.

The location of the double bond of the major acids was determined by dimethyl disulfide (DMDS) derivatization [32,34]. The fragment ions at *m*/*z* 145 and 161 of the DMDS-derivative and the secondary fragments obtained by loss of the methyl ester group (*m*/*z* 129) located the position of the double bond in C12:1-FAME at C-5. Similarly, 9-C16:1 (*m*/*z* 145, 185, 217) and 11-C18:1-FAMEs (*m*/*z* 145, 213, 245) were assigned. The three *Phaeobacter* strains showed also a small peak with identical mass spectrum compared to 11-C18:1-FAME eluting slightly earlier than the major compound, indicating minor amounts of 11*E*-C18:1-FAME next to the major 11*Z-*C18:1-FAME. DMDS adducts derived from *E*-configured double bonds elute slightly earlier than their *Z*-configured counterparts on apolar GC phases [34]. All four strains additionally contained 13-C18:1-FAME (*m*/*z* 61, 117, 241 and 273) in small amounts. The mass spectrum of C19:1-FAME differed from that of methyl nonadecenoate, but was identical to that of methyl 11-methyl-12-octadecenoate [29,35–36], as was that of its DMDS derivative (*m*/*z* 131, 241, 273, see Figures S1 and S2 in the Supporting Information File 1). Similarly, 13-C20:1 was identified in *D. shibae* DFL-12. Small amounts of a DMDS adduct of C18:2 were detected that added only one equivalent of DMDS. This reactivity is observed when a double bond is conjugated with a carbonyl group [37–38]. The ion at *m*/*z* 145 located one double bond at C-11, while the ions at *m*/*z* 211 and 243 revealed another unsaturation in the alkyl chain towards the carboxy terminus. These data indicate this FAME to be 2,11-C18:1, the parent acid of the major *D. shibae* AHL, 2*E*,11*Z-*C18:2-HSL [10]. The analysis performed with bacteria grown in liquid medium led to comparable results, indicating that the fatty acid composition does not depend on the culture method.

By comparing the fatty acid profiles and AHL production no direct correlation between fatty acids and AHLs can be observed. The major acid C18:1 is only reflected by a minor component in the AHL profile of the four strains. Small amounts of 2,11-C18:2 occur in all strains, only the *D. shibae* strain uses this acid as precursor for its major 2,11-C18:2-HSL. In contrast, the precursor acid 3-OH-C10:0-HSL is produced by *D. shibae*, but not present in the profiles of *P. inhibens*, which produces large amounts of 3-OH-C10:0-HSL. Furthermore, the prominent acid 11Me-12-C18:1 is not used for AHL formation. Acids used for production of minor AHLs such as C14:1 or C12:2 were not detected.

These results show that the fatty acid pool and AHL formation are indeed uncoupled. Although the fatty acid composition of the investigated strains is very similar, the AHL production differs largely. The complete absence or presence of only minor amounts of precursor acids of AHLs such as 2,11-C18:2 or 3-OH-C10:0-HSL might indicate that they are available only for AHL biosynthesis, but are not used for other physiological purposes. Such acids may be immediately transformed after their biosynthesis into an AHL, or are stored in a form not cleavable by the TMSH method used. These precursor acids may also originate from fatty acid degradation, a pathway that proceeds via free coenzyme A intermediates and not via acyl carrier protein-bound substrates like in the fatty acid biosynthesis.

**Scheme 2:**
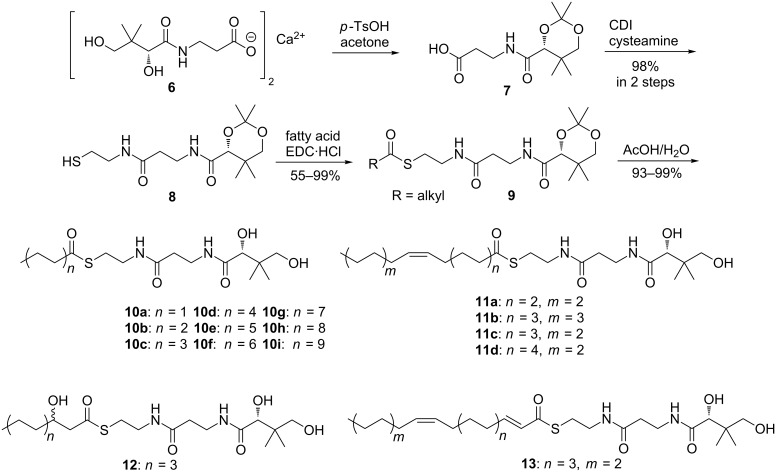
Synthesis of *N*-pantothenoylcysteamine thioesters (PCEs) for feeding experiments with AHL synthases.

These results led to the question whether the acyl-chain selectivity is an inherent property of the AHL synthase itself or whether this is determined by other factors, e.g., precursor availability. Therefore, LuxI-type synthases from *D. shibae* (LuxI_1_, LuxI_2_) and from *P. inhibens* DSM17395 (PgaI_2_) were cloned and expressed in *E. coli* to allow in vitro experiments with suitable acyl precursors to probe AHL formation. After protein purification of the AHL synthases and incubation with the precursors *S*-adenosyl methionine (SAM) and different acyl derivatives (free fatty acids, SNAC esters, PCEs) the AHL production was determined using GC/MS [9–10,32]. Coenzyme A or abbreviated ACP analogs, *N*-pantothenoylcysteamine thioesters of fatty acids (PCEs) were synthesized (Scheme 2) to serve as substrate substitutes for the native precursors.

Calcium pantothenate (**6**) was protected with acetone forming acid **7** that was transformed with cysteamine into the protected thiol **8** [39]. Steglich esterification [40] with different free acids led to nine saturated PCEs **10a**–**i**, four monounsaturated acids (**11a**–**d**), 3-OH-C10:0-HSL PCE (**12**), and 2*E*,11*Z*-C18:2-PCE (**13**) after deprotection with acetic acid [41]. Although compounds **10**–**13** can be further purified by HPLC, the crude products proved to be pure enough for the next experiments.

The incubation experiments were performed with the three recombinant AHL synthases, SAM, and the different precursors **10**–**13**. The AHL-synthase PgaI_2_ of *P. inhibens* showed a higher activity compared to the two *D. shibae* synthases. It accepted all substrates, including unsaturated ones, with the exclusion of the very short C4:0 and very long C20:0-PCEs.

The AHL-synthase LuxI_2_ was able to produce C14:0-HSL and 3-OH-C10-HSL in low concentration from the respective precursors. It is likely responsible for the formation of C14:0-HSL and 3-oxo-C14:0-HSL in *D. shibae* DFL-12. The AHL synthase LuxI_1_ used five precursors to synthesize C8:0, C10:0, C12:0 and C14:0-HSL in low amounts, while 2*E*,11*Z-*C18:2-HSL, its native product, is formed in high concentration.

**Figure 2:**
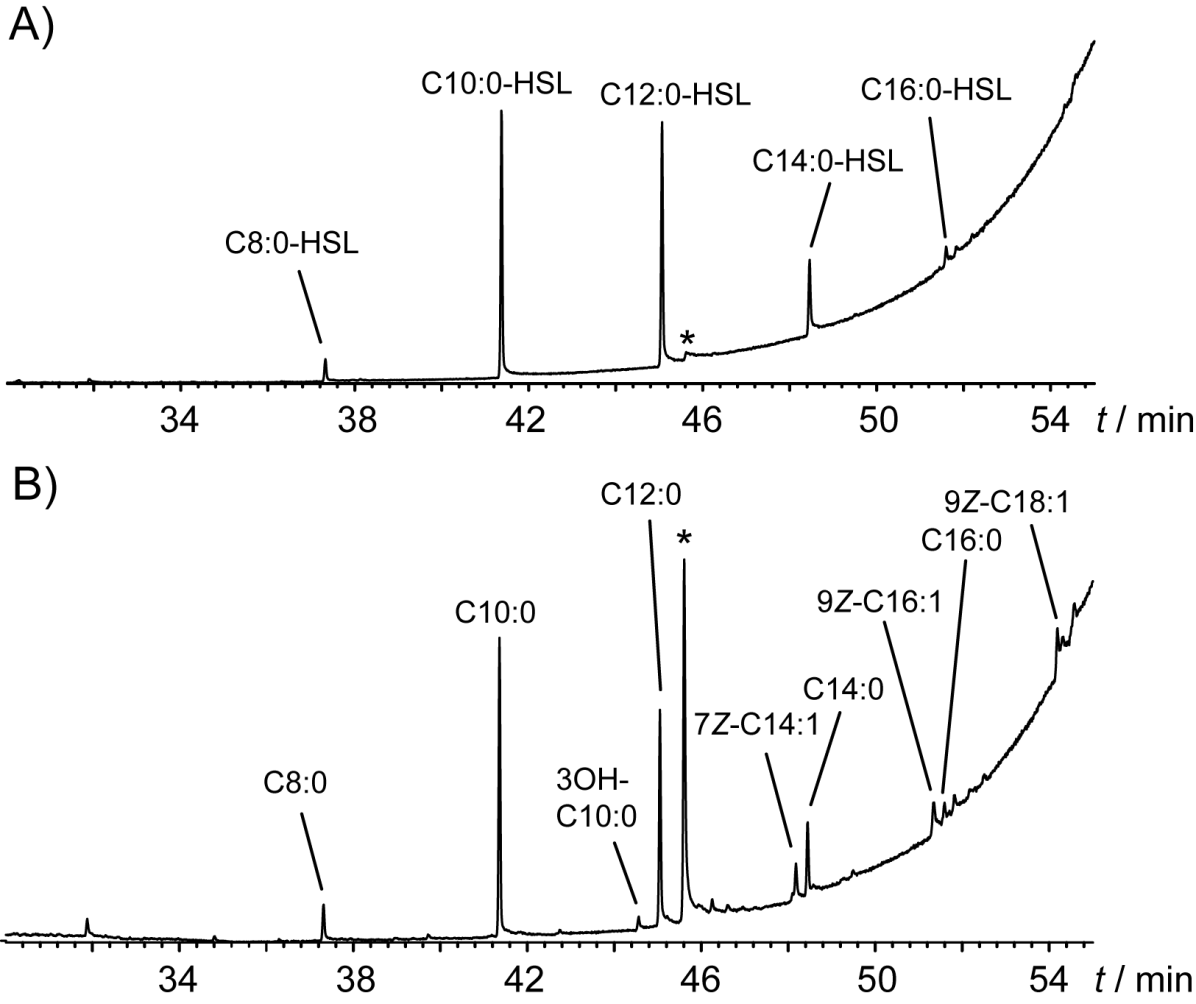
Total ion chromatograms of the extracts from competition experiments using recombinant PgaI_2_ from *P. inhibens* with SAM and a mixture of equally concentrated substrates A) **10a**–**h** (saturated chains C_4_–C_18_) and B) **10a**–**i**, **11a**–**c** and **12**. Prominent contaminants are indicated by asterisks.

To further evaluate the selectivity of the promiscuous enzyme PgaI_2_ from *P. inhibens*, competition experiments were performed. Targeting the optimal chain length of the fully saturated substrates first, a mixture with equal molar concentrations of the substrates **10a**–**h** was offered to the recombinant protein. GC/MS analysis of the resulting extract (Figure 2A) revealed a distribution of AHL products around the chain length of C_10_ and C_12_, which were shown to be the most prominent products. In a second experiment with substrates **10a**–**i**, **11a**–**c**, and **12** also unsaturated substrates and the hydroxylated precursor were tested (Figure 2B). It turned out that the same distribution of the saturated AHLs as for the first experiment was observed with none of the additional substrates showing a significantly higher conversion. These results point to a very flexible active site of the investigated AHL synthase PgaI_2_, which converts a variety of substrates. The highest conversion efficiency in the competition experiments was found for the saturated substrates **10c** and **10d** with lower abundance of any AHL products deviating from this chain length. It should be noted that the amount of added SAM was not sufficient to convert all substrates, so the product spectrum likely reflects different enzyme kinetics for the PCE substrates. In contrast, in the single-substrate incubation experiments (Table 3) an excess of SAM was used, and this may have led to the formation even of products that are disfavored in the competition experiments.

**Table 3:** Results of incubation experiments of single precursors **10**–**13** with *E. coli* constructs with recombinant AHL synthases PgaI_2_, LuxI_1_ and LuxI_2_ from different *Roseobacter* group bacteria.^a^

Precursor	AHL	*P. inhibens* PgaI_2_	*D. shibae* LuxI_1_	*D. shibae* LuxI_2_

**10a**	C4:0	–	–	–
**10b**	C6:0	x	–	–
**10c**	C8:0	xx	x	–
**10d**	C10:0	xx	x	–
**10e**	C12:0	xx	x	–
**10f**	C14:0	xx	x	x
**10g**	C16:0	x	–	–
**10h**	C18:0	x	–	–
**10i**	C20:0	–	–	–
**12**	3-OH-C10:0	xx	–	x
**11a**	7*Z*-C14:1	xx	–	–
**11b**	9*Z*-C16:1	xx	–	–
**11c**	9*Z*-C18:1	xx	–	–
**11d**	11*Z*-C18:1	xx	–	–
**13**	2*E*,11*Z*-C18:2	xx	xx	–

^a^xx: high production, x: low production, –: no production.

## Conclusion

The results showed that the enzymes exhibit varying substrate plasticity. While the *P. inhibens* synthase PgaI_2_ accepted most precursors, the best performance was observed with the saturated substrates harboring C_10_ or C_12_ chain lengths. In *P. inhibens* this enzyme is most likely responsible for the biosynthesis of long-chain AHLs. In contrast, *D. shibae* synthase LuxI_1_ showed a high selectivity for 2*E*,11*Z-*C18:2-HSL and did not even accept similar substrates such as **11c** or **11d**. Interestingly, considerably shorter saturated substrates, e.g., **10e**, are accepted. The *D. shibae* synthase LuxI_2_ synthase was even more selective. It seems likely that other factors than AHL synthase substrate specificity influence the observed formation of only certain AHLs by these wild-type enzymes. These factors might include selectivity found in enzymes activating or transporting acids to AHL synthases, or interact with the LuxI enzyme, either directly or indirectly. The combination of the different selectivity levels may eventually lead to the specific mixtures observed in the different AHL producing bacterial strains.

## Supporting Information

File 1 Experimental, mass spectra, SDS page and NMR spectra.
