# Supplementary material for: Acyl-group specificity of AHL synthases involved in quorum-sensing in Roseobacter group bacteria
Source: Beilstein J Org Chem. 2018 Jun 5;14:1309–16. doi: 10.3762/bjoc.14.112 (PMC6009203; doi:10.3762/bjoc.14.112)
Supplement: File 1 — Experimental, mass spectra, SDS page and NMR spectra. [file Beilstein_J_Org_Chem-14-1309-s001.pdf]

Supporting Information

for

Acyl-group specificity of AHL synthases involved in quorum-sensing in *Roseobacter* group bacteria

Lisa Ziesche[1], Jan Rinkel[2], Jeroen S. Dickschat[2] and Stefan Schulz[*1]

Address: [1]Institute of Organic Chemistry, Technische Universität Braunschweig, Hagenring 30, 38106 Braunschweig, Germany and [2]Kekulé-Institute of Organic Chemistry and Biochemistry, University of Bonn, Gerhard-Domagk-Str. 1, 53121 Bonn, Germany

Email: Stefan Schulz - stefan.schulz@tu-bs.de

*Corresponding author

Experimental, mass spectra, SDS page and NMR spectra

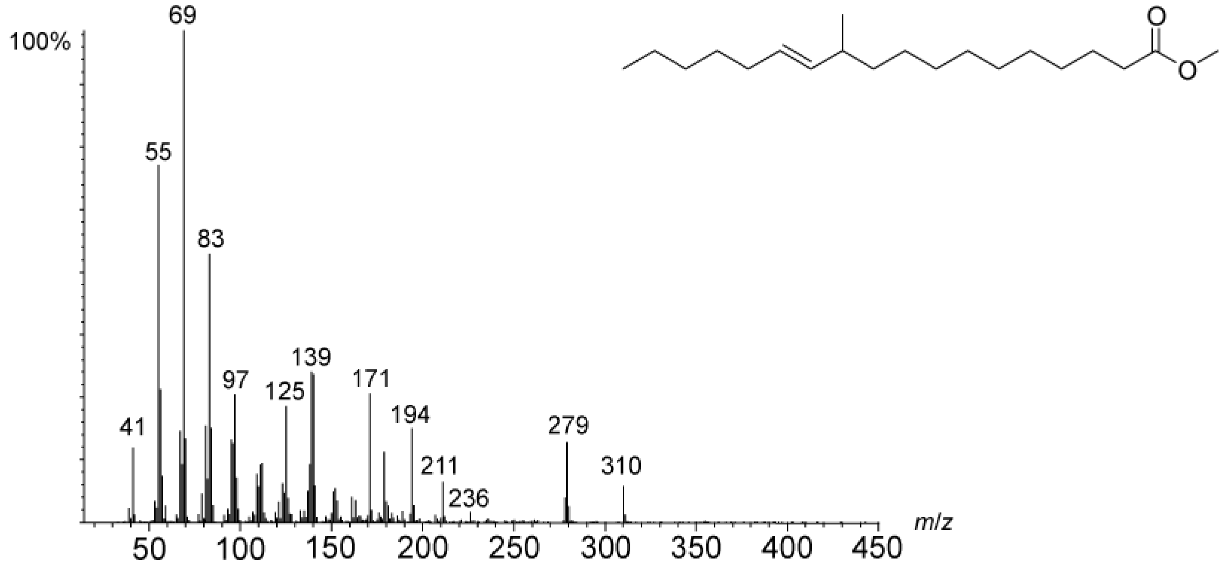

Figure S1: EI-mass spectrum of methyl (*E*)-11-methyloctadec-12-enoate.

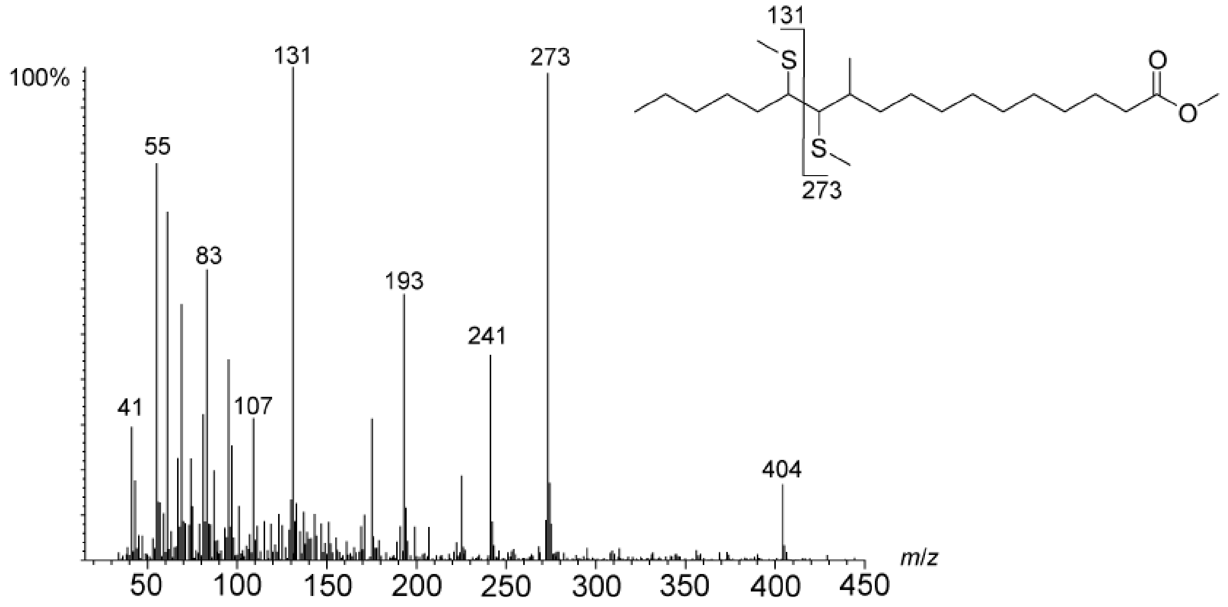

Figure S2: EI-mass spectrum of the dimethyl disulfide adduct of methyl (*E*)-11-methyloctadec-12-enoate.

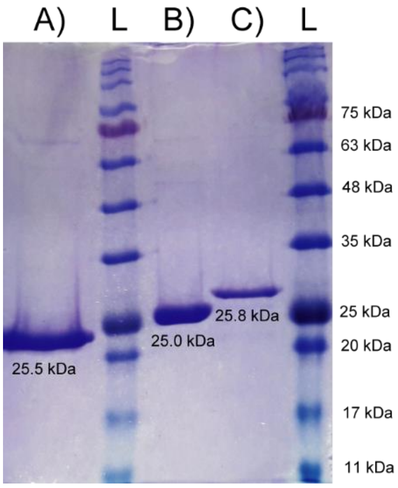

Figure S3: SDS-PAGE analysis of obtained elution fractions of A) $PgaI_2$, B) $LuxI_1$, and C) $LuxI_2$. Calculated molecular weights of the recombinant proteins are shown.

Experimental

General conditions: Chemicals were obtained from Sigma-Aldrich, Carl Roth or from Acros Organics and were used without further purification. Solvents were purified by distillation and dried according to standard procedures. Moisture and/or oxygen-sensitive reactions were carried out under a nitrogen atmosphere in vacuum-heated flasks with dried solvents. Thin layer chromatography (SiO_2, TLC) was performed on 0.20 mm Macherey-Nagel silica gel plates (Polygram SIL G/UV254), and column chromatography was performed with Merck silica gel 60 (0.040–0.063 mm) by using standard flash chromatographic methods. NMR spectra were recorded on Bruker DRX-400 (400 MHz), AV III-400 (400 MHz), AV III-HD500 (500 MHz) or AV II-600 (600 MHz) spectrometers and were referenced against TMS (δ = 0.00 ppm), $CDCl_3$ (δ = 7.26 ppm), and CD_3OD (δ = 3.32 ppm) for ^1H NMR and $CDCl_3$ (δ = 77.01 ppm) and CD_3OD (δ = 49.0 ppm) for ^{13}C NMR experiments. GC/MS analyses of extracts were carried out on an Agilent GC 7890A gas chromatograph connected to a 5975C mass-selective detector (Agilent). Synthetic samples were analyzed on an HP GC 6890 system connected to a HP 5973 mass selective detector fitted with a HP-5

MS fused-silica capillary column (30 m × 0.25 mm i.d., 0.22 µm film; Hewlett-Packard). Conditions were as follows: carrier gas (He): 1.2 mL min^{-1}; injection volume: 1 mL; injector: 250 °C; transfer line: 300 °C, EI 70 eV. The gas chromatograph was programmed as follows: 50 °C (5 min isothermal), increasing with 5 °C min^{-1} to 320 °C, and operated in splitless mode for extracts and 50 °C (5 min isothermal), increasing with 10 °C min^{-1} to 320 °C in split mode (20:1) for synthetic compounds. Gas chromatographic retention indices, *I*, were determined from a homologous series of *n*-alkanes. HPLC/MS analyses were carried out on a Thermo Fisher Accela LC System connected to an LTQ XL mass spectrometer (Thermo Scientific) operated in the ESI positive mode and fitted with a RP-C18 column (150 mm length, 2.1 mm diameter, Agilent). HRMS analyses were carried out on a Thermo Fisher linear iontrap coupled with a LTQ-Orbitrap mass spectrometer in the ESI positive mode. ESI measurements were performed by direct infusion mode using a custom made microspray device mounted on a Proxeon nanospray ion source. All solvents used were of LC/MS grade.

Strains and culture conditions: *Phaeobacter inhibens* DSM17395 and *Dinoroseobacter shibae* DFL-12 were obtained from the DSMZ collection (Braunschweig, Germany) and cultured in marine broth medium (MB) at 28 °C.

XAD extracts: Bacterial cultures (100 mL) were grown for three days in marine broth medium containing Soxhlet precleaned Amberlite XAD-16 (2 g). The adsorbent was separated from the culture by filtration and extracted three times with CH_2Cl_2/H_2O (10:1) [1]. The combined organic phases were dried with $MgSO_4$ and the solvent was removed under reduced pressure. The extract was redissolved in CH_2Cl_2 (50 µL) and analyzed by GC/MS.

Fatty acid methyl esters: A few colonies of bacteria were transferred with a loop from an agar plate or a cell pellet, obtained by centrifugation from a well-grown liquid culture, to a 1 mL GC vial filled with 10 µL distilled water. The bacteria were directly lysed by adding 30 µL methanolic trimethylsulfonium hydroxide (TMSH) solution (0.25 M) that concomitantly converted bound and free fatty acids to FAMEs [2]. The mixture was dried in a nitrogen stream and the residue was dissolved in 200 µL diethyl ether/methanol (10:1). This solution was analyzed by a GC/MS.

Determination of double bond positions: FAME samples were dissolved in 50 µL dichloromethane (DCM) and 50 µL of dimethyl disulfide in a 1 mL vial, and 5 µL of 5% ethereal iodine solution was added. The closed vial was kept for a day in a heating block at 60 °C. The cooled mixture was diluted with 200 µL of DCM. The mixture was shaken with 200 µL of aqueous sodium sulfite solution to reduce iodine. After separation of the phases, the organic phase was dried with $MgSO_4$, filtered and the solvent was evaporated in a gentle stream of nitrogen. The residue was redissolved in DCM and analyzed by GC/MS [1,3,4].

Gene cloning: Genomic DNA was isolated from liquid cultures of *P. inhibens* DSM17395 and *D. shibae* DFL-12 in half strength MB medium grown for 7 days using a standard protocol based on phenol/chloroform/isoamyl alcohol extraction [5]. Using these templates, the target AHL synthase genes *pgaI*$_2$, *luxI*$_1$ and *luxI*$_2$ (for accession numbers, see Table S1) were amplified by PCR using Phusion DNA polymerase (New England Biolabs, Ipswich, MA, USA) and the primers without homology arms listed in Table 5 (PCR conditions: initial denaturation 95 °C for 5 min; 30 cycles 95 °C for 15 s, annealing 68 °C for 30 s, 72 °C for 30 s; final elongation 72 °C for 5 min). The obtained fragments were further elongated in a second PCR

reaction using the corresponding primers harboring homology arms (Table S2) with the reaction conditions described above. These fragments were added to linearized yeast-*E. coli* shuttle vector pYE-express [6] (HindIII and EcoRI digested) and used for homologous recombination in yeast by the LiOAc/SS DNA/PEG protocol [7]. Transformed yeast colonies were grown on SM-URA medium to isolate the product plasmids by Plasmid Miniprep II kit (Zymo Research, Irvine, USA). The obtained plasmid mixtures were shuttled to *E. coli* BL21(DE3) cells by electroporation. The transformed cells were grown on selective LB agar medium (10 g/L tryptone, 5 g/L yeast extract, 5 g/L NaCl, 18 g/L agar, pH 7.2) supplied with kanamycin (50 μg/mL) to pick single colonies, which were grown in liquid LB medium (50 μg/mL kanamycin) at 37 °C. Isolated plasmids were checked for correct insertion of the target gene by analytical digest and sequencing of the positive samples.

Table S1: AHL synthases from *P. inhibens* DSM17395 and *D. shibae* DFL-12.

Organism	Abbreviation[a]	Locus tag	Accession nr.	Alt. accession nr.
P. inhibens DSM17395	$\mathrm{PgaI_1}$	PGA1_c03890	WP_014879070	ZP_02146368, AFO90123
P. inhibens DSM17395	**$\mathbf{PgaI_2}$**	PGA1_c07680	WP_014879372	AFO90496
D. shibae DFL-12	**$\mathbf{LuxI_1}$**	Dshi_0312	WP_012176991	ABV92061
D. shibae DFL-12	**$\mathbf{LuxI_2}$**	Dshi_2851	WP_012179512	ABV94584
D. shibae DFL-12	$\mathrm{LuxI_3}$	Dshi_4152	WP_012187430	ABV95863

[a]Enzymes investigated in this work are shown in bold. Abbreviations are used in analogy to previous work [1,8,9].

Table S2: Primers used for gene cloning.

Primer	Sequence[a]
SK001f_AFO90496	ATGCAGTCGACAGAAATTACATTCG
SK001r_AFO90496	TTATGCTGCGTCTGCGTCG
SK002f_AFO90496	GGCAGCCATATGGCTAGCATGACTGGTGGAATGCAGTCGACAGAAATTACATTCG
SK002r_AFO90496	TCTCAGTGGTGGTGGTGGTGGTGCTCGAGTTTATGCTGCGTCTGCGTCG
SK005f_WP012176991	ATGCAAACCACCACGCTTTCG
SK005r_WP012176991	CTAGTGCAGCTTGCGGGTCAGG
SK006f_WP012176991	GGCAGCCATATGGCTAGCATGACTGGTGGAATGCAAACCACCACGCTTTCG
SK006r_WP012176991	TCTCAGTGGTGGTGGTGGTGGTGCTCGAGTCTAGTGCAGCTTGCGGGTCAGG
SK007f_WP012179512	ATGATCCGTTTCGTCTATGCCGACC
SK007r_WP012179512	TCAGGCCGCCAAGCTGGG
SK008f_WP012179512	GGCAGCCATATGGCTAGCATGACTGGTGGAATGATCCGTTTCGTCTATGCCGACC
SK008r_WP012179512	TCTCAGTGGTGGTGGTGGTGGTGCTCGAGTTCAGGCCGCCAAGCTGGG

[a]Homology arms for recombination in yeast fitting to pYE-express are underlined.

Gene expression and protein purification: The *E. coli* BL21(DE3) transformants harboring the corresponding expression plasmids were grown in LB medium (10 g/L tryptone, 5 g/L yeast extract, 5 g/L NaCl, pH 7.2) supplied with kanamycin (50 μg/mL) at 37 °C overnight. The precultures were used to inoculate main cultures using the same medium (250 mL), which were grown at 37 °C until OD_{600} 0.4–0.6 was reached. The cultures were cooled to 18 °C and expression was induced by adding isopropyl-β-D-thiogalactopyranosid (IPTG) to a final concentration of 400 μM. The cultures were further incubated at 18 °C for 16 h, before the cells were harvested by centrifugation (5000*g*, 10 min, 4 °C) and resuspended in binding buffer (5 mL; 50 mM TRIS-HCl, 500 mM NaCl, 20 mM imidazole, pH 8.0, 4 °C). Cell disruption was done

by ultra-sonication on ice (50% power, 5 × 30 s, 4 °C) and the cell debris was removed by centrifugation (5500*g*, 10 min, 4 °C) to yield the soluble protein fraction, which was applied to Ni^{2+}-NTA affinity chromatography using Ni-NTA superflow (Qiagen, Venlo, Netherlands). Washing was done by binding buffer (3 × 2 mL) and the target protein was isolated by elution buffer (2 × 1 mL; 50 mM TRIS-HCl, 500 mM imidazole, 20% glycerol, pH 8.0, 4 °C). The obtained protein solution was analyzed by SDS-PAGE for the target protein size (Figure S3) and used for incubation experiments.

Incubation experiments with recombinant AHL synthases: For substrate testing, each synthetic substrate analogue (1 μmol) was dissolved in DMSO (30 μL) and diluted by binding buffer (470 μL). After adding glycerol (50 μL), an AHL synthase elution fraction (250 μL) was supplied to the mixture and the reaction was started by adding a solution of *S*-adenosylmethionine chloride dihydrochloride (SAM, 0.67 mg, 1 μmol, in 200 μL H_2O). The reaction was incubated for 5 h at 28 °C before it was extracted with DCM (200 μL). The extract was washed with water (200 μL), dried with $MgSO_4$, filtered and analyzed by GC/MS for the target AHLs.

For the competition experiments, a solution of the mixed substrate analogues **10a**–**h** or **10a**–**i**, **11a**–**c** and **12** (0.1 μmol each, in 50 μL DMSO) was added to binding buffer (450 mL) and glycerol (50 mL), before AHL synthase elution fraction (250 mL) and finally a solution of SAM (1.1 mg, 1.6 μmol, in 200 μL H_2O) were added. The reaction was incubated, extracted and analyzed as described above.

(*R*)-*N*-Pantothenoylcysteamine acetonide (8)

Calcium (*R*)-pantothenate (5 g, 10.49 mmol), *p*-toluenesulfonic acid monohydrate (4.79 g, 25.18 mmol), 3 Å molecular sieves (5 g) and 250 mL HPLC grade acetone were stirred overnight at room temperature in a round-bottomed flask. The suspension was filtered through Celite, washed three times with 100 mL acetone and the solvent was evaporated. The oil was redissolved in 200 mL EtOAc, washed two times with 100 mL brine and dried with Na_2SO_4. Most of the solvent was removed and hexane was added slowly to form a white solid. The solid was dried in high vacuum and used in the next step without further purification.

The freshly prepared ketal **7** (5.06 g, 19.51 mmol) was dissolved in 100 mL dry THF, treated with 1,1'-carbonyldiimidazole (CDI) (4.75 g, 29.27 mmol) and stirred for one hour at room temperature. Cysteamine hydrochloride (3.38 g, 29.27 mmol) was added to this solution and the mixture stirred at room temperature overnight. THF was removed in vacuo and the residue was redissolved in DCM. The organic phase was washed with 50 mL sat. NH_4Cl solution and with 50 mL brine, dried with Na_2SO_4, filtered, and concentrated. The crude product was purified by flash chromatography on silica (EtOAc/pentane 70:30 to 100% EtOAc) to get the thiol as a white solid (6.1 g, 19.15 mmol, 98%) [10].

R_f = 0.2 (EtOAc/pentane 70:30); ^{1}H NMR (400 MHz, $CDCl_3$): δ = 7.04 (br s, 1H), 6.49 (br s, 1H), 4.08 (s, 1H), 3.69 (d, *J* = 11.8 Hz, 1H), 3.63-3.51 (m, 2H), 3.51-3.36 (m, 2H), 3.29 (d, *J* = 11.8 Hz, 1H), 2.69-2.63 (m, 2H), 2.48 (t, *J* = 6.3 Hz, 2H), 1.46 (s, 3H), 1.42 (s, 3H), 1.04 (s, 3H), 0.97 (s, 3H); ^{13}C NMR (100 MHz, $CDCl_3$): δ = 171.1, 170.2, 99.1, 77.1, 71.4, 42.4, 36.1, 34.8, 32.9, 29.4, 24.5, 22.1, 18.9, 18.7; MS (70 eV, EI): *m*/*z* (%): 59 (100), 143 (97), 260 (69), 88 (69), 206 (55), 43 (50), 55 (32), 245 (27), 184 (25), 303 (24), 318 [M^+].

(*Z*)-7-Tetradecenoic acid

To a solution of 7-tetradecynoic acid (0.22 g, 0.98 mmol) and methanol (5 mL) Lindlar's catalyst (0.02 g) was added. The mixture was stirred for 20 minutes at room temperature under a hydrogen atmosphere. The catalyst was removed by filtration through a short pad of silica and the solvent was evaporated. The product was obtained as colorless oil without the need of further purification (205 mg, 0.90 mmol, 92%) [11]. All analytical data were in agreement with those previously reported [4].

***S*-Acyl-*N*-pantothenoyl acetonide derivatives (9)**

To a stirred solution of an acid in dry DCM, *p*-dimethylaminopyridine (DMAP) and **8** were added. *N*-(3-Dimethylaminopropyl)-*N*'-ethylcarbodiimide hydrochloride (EDC·HCl) was added at 0 °C, the solution was stirred for 5 minutes at 0 °C and overnight at room temperature [12]. 1N HCl was added to the reaction mixture followed by extraction with DCM three times. The combined organic phases were washed with sat. $NaHCO_3$ sol. and brine, dried with $MgSO_4$, filtered and concentrated to give an oil.

(*R*)-*S*-Butanoyl-*N*-pantothenoylcysteamine acetonide

Colorless oil (435 mg, 1.12 mmol, 99%) R_f = 0.3 (EtOAc/pentane 6:1); ^1H NMR (400 MHz, $CDCl_3$): δ = 7.03 (br s, 1H), 6.24 (br s, 1H), 4.08 (s, 1H), 3.69 (d, *J* = 11.5 Hz, 1H), 3.62-3.48 (m, 2H), 3.48-3.38 (m, 2H), 3.28 (d, *J* = 11.8 Hz, 1H), 3.01 (t, *J* = 6.5 Hz, 2H), 2.57-2.53 (m, 2H), 2.43 (t, *J* = 6.3 Hz, 2H), 1.74-1.65 (m, 2H), 1.46 (s, 3H), 1.42 (s, 3H), 1.26 (t, *J* = 7.0 Hz, 3H), 1.04 (s, 3H), 0.97 (s, 3H); ^{13}C NMR (100 MHz, $CDCl_3$): δ = 199.7, 171.1, 170.1, 99.1, 77.1, 71.4, 45.9, 39.6, 35.9, 34.8, 33.0, 29.4, 28.4, 22.1, 19.1, 18.9, 18.7, 13.4; (+)-ESI MS *m/z*: 389.3 $[M+H]^+$, 411.3 $[M+Na]^+$.

(*R*)-*S*-Hexanoyl-*N*-pantothenoylcysteamine acetonide

Colorless oil (354 mg, 0.85 mmol, 98%) R_f = 0.4 (EtOAc/pentane 6:1); ^{1}H NMR (400 MHz, $CDCl_3$): δ = 7.03 (br s, 1H), 6.20 (br s, 1H), 4.08 (s, 1H), 3.69 (d, *J* = 11.8 Hz, 1H), 3.61-3.53 (m, 2H), 3.51-3.38 (m, 2H), 3.28 (d, *J* = 11.8 Hz, 1H), 3.01 (t, *J* = 6.3 Hz, 2H), 2.56 (t, *J* = 7.5 Hz, 2H), 2.42 (t, *J* = 6.3 Hz, 2H), 1.70-1.62 (m, 2H), 1.46 (s, 3H), 1.42 (s, 3H), 1.35-1.29 (m, 2H), 1.28-1.24 (m, 2H), 1.04 (s, 3H), 0.97 (s, 3H), 0.89 (t, *J* = 7.0 Hz, 3H); ^{13}C NMR (100 MHz, $CDCl_3$): δ = 199.8, 171.1, 170.1, 99.1, 77.2, 71.4, 44.1, 39.6, 35.9, 34.8, 33.0, 31.1, 29.5, 28.4, 25.3, 22.3, 22.1, 18.9, 18.7, 13.8; (+)-ESI MS *m/z*: 417.3 $[M+H]^+$, 439.3 $[M+Na]^+$.

(*R*)-*S*-Octanoyl-*N*-pantothenoylcysteamine acetonide

Colorless oil (315 mg, 0.70 mmol, 99%) R_f = 0.4 (EtOAc/pentane 6:1); ^{1}H NMR (400 MHz, $CDCl_3$): δ = 7.03 (br s, 1H), 6.13 (br s, 1H), 4.08 (s, 1H), 3.69 (d, *J* = 11.8 Hz, 1H), 3.61-3.50 (m, 2H), 3.50-3.38 (m, 2H), 3.29 (d, *J* = 11.8 Hz, 1H), 3.01 (t, *J* = 6.5 Hz, 2H), 2.56 (t, *J* = 7.5 Hz, 2H), 2.42 (t, *J* = 6.3 Hz, 2H), 1.69-1.62 (m, 2H), 1.46 (s, 3H), 1.42 (s, 3H), 1.30-1.24 (m, 8H), 1.04 (s, 3H), 0.97 (s, 3H), 0.88 (t, *J* = 6.8 Hz, 3H); ^{13}C NMR (100 MHz, $CDCl_3$): δ = 199.9, 171.1, 170.1, 99.1, 77.2, 71.5, 44.1, 39.6, 36.0, 34.8, 33.0, 31.6, 29.5, 28.9, 28.9, 28.4, 25.6, 22.6, 22.1, 18.9, 18.7, 14.0; (+)-ESI MS *m/z*: 445.3 $[M+H]^+$, 467.3 $[M+Na]^+$.

(*R*)-*S*-Decanoyl-*N*-pantothenoylcysteamine acetonide

Colorless oil (224 mg, 0.47 mmol, 86%) R_f = 0.4 (EtOAc/pentane 6:1); ^{1}H NMR (400 MHz, $CDCl_3$): δ = 7.03 (br s, 1H), 6.13 (br s, 1H), 4.08 (s, 1H), 3.69 (d, *J* = 11.8 Hz, 1H), 3.61-3.50 (m, 2H), 3.50-3.38 (m, 2H), 3.29 (d, *J* = 11.8 Hz, 1H), 3.01 (t, *J* = 6.5 Hz, 2H), 2.56 (t, *J* = 7.3 Hz, 2H), 2.42 (t, *J* = 6.3 Hz, 2H), 1.70-1.61 (m, 2H), 1.46 (s, 3H), 1.42 (s, 3H), 1.33-1.24 (m, 12H), 1.04 (s, 3H), 0.97 (s, 3H), 0.88 (t, *J* =

6.5 Hz, 3H); ^{13}C NMR (100 MHz, $CDCl_3$): δ = 199.9, 171.1, 170.1, 99.1, 77.2, 71.5, 44.1, 39.6, 36.0, 34.8, 33.0, 31.8, 29.5, 29.3, 29.2, 29.2, 28.9, 28.4, 25.6, 22.6, 22.1, 18.9, 18.7, 14.1; (+)-ESI MS *m/z*: 473.3 $[M+H]^+$, 495.3 $[M+Na]^+$.

(*R*)-*S*-Dodecanoyl-*N*-pantothenoylcysteamine acetonide

Colorless oil (250 mg, 0.49 mmol, 98%) R_f = 0.4 (EtOAc/pentane 6:1); ^{1}H NMR (400 MHz, $CDCl_3$): δ = 7.03 (br s, 1H), 6.18 (br s, 1H), 4.08 (s, 1H), 3.69 (d, *J* = 11.5 Hz, 1H), 3.61-3.53 (m, 2H), 3.51-3.39 (m, 2H), 3.28 (d, *J* = 11.8 Hz, 1H), 3.01 (t, *J* = 6.5 Hz, 2H), 2.56 (t, *J* = 7.5 Hz, 2H), 2.42 (t, *J* = 6.3 Hz, 2H), 1.68-1.61 (m, 2H), 1.46 (s, 3H), 1.42 (s, 3H), 1.33-1.24 (m, 16H), 1.04 (s, 3H), 0.97 (s, 3H), 0.88 (t, *J* = 6.8 Hz, 3H); ^{13}C NMR (100 MHz, $CDCl_3$): δ = 199.9, 171.1, 170.1, 99.1, 77.1, 71.4, 44.1, 39.6, 35.9, 34.8, 33.0, 31.9, 29.6, 29.6, 29.5, 29.4, 29.3, 29.2, 28.9, 28.4, 25.6, 22.7, 22.1, 18.9, 18.7, 14.1; (+)-ESI MS *m/z*: 501.3 $[M+H]^+$, 523.3 $[M+Na]^+$.

(*R*)-*S*-Tetradecanoyl-*N*-pantothenoylcysteamine acetonide

Colorless oil (227 mg, 0.43 mmol, 98%) R_f = 0.4 (EtOAc/pentane 5:1); ^{1}H NMR (400 MHz, $CDCl_3$): δ = 7.03 (br s, 1H), 6.13 (br s, 1H), 4.08 (s, 1H), 3.69 (d, *J* = 11.5 Hz, 1H), 3.64-3.50 (m, 2H), 3.50-3.38 (m, 2H), 3.28 (d, *J* = 11.8 Hz, 1H), 3.01 (t, *J* = 6.5 Hz, 2H), 2.56 (t, *J* = 7.5 Hz, 2H), 2.42 (t, *J* = 6.3 Hz, 2H), 1.69-1.61 (m, 2H), 1.46 (s, 3H), 1.42 (s, 3H), 1.33-1.24 (m, 20H), 1.04 (s, 3H), 0.97 (s, 3H), 0.88 (t, *J* = 6.5 Hz, 3H); ^{13}C NMR (100 MHz, $CDCl_3$): δ = 199.9, 171.1, 170.1, 99.1, 77.2, 71.5, 44.2, 39.6, 36.0, 34.8, 33.0, 31.9, 29.7, 29.6, 29.6, 29.6, 29.5, 29.4, 29.3, 29.2, 28.9, 28.4, 25.6, 22.7, 22.1, 18.9, 18.7, 14.1; (+)-ESI MS *m/z*: 529.3 $[M+H]^+$, 551.4 $[M+Na]^+$.

(*R*)-*S*-Hexadecanoyl-*N*-pantothenoylcysteamine acetonide

Colorless oil (211 mg, 0.38 mmol, 97%) R_f = 0.4 (EtOAc/pentane 4:1); ^{1}H NMR (400 MHz, $CDCl_3$): δ = 7.02 (br s, 1H), 6.11 (br s, 1H), 4.08 (s, 1H), 3.69 (d, *J* = 11.5 Hz, 1H), 3.64-3.51 (m, 2H), 3.50-3.36 (m, 2H), 3.28 (d, *J* = 11.5 Hz, 1H), 3.01 (t, *J* = 6.5 Hz, 2H), 2.56 (t, J= 7.5 Hz, 2H), 2.42 (t, *J* = 6.1 Hz, 2H), 1.69-1.61 (m, 2H), 1.46 (s, 3H), 1.42 (s, 3H), 1.34-1.24 (m, 24H), 1.04 (s, 3H), 0.97 (s, 3H), 0.88 (t, *J* = 6.5 Hz, 3H); ^{13}C NMR (100 MHz, $CDCl_3$): δ = 199.9, 171.1, 170.1, 99.1, 77.2, 71.5, 44.2, 39.6, 36.0, 34.8, 33.0, 31.9, 29.7, 29.7, 29.7, 29.6, 29.6, 29.6, 29.5, 29.4, 29.4, 29.2, 29.0, 28.4, 25.6, 22.7, 22.1, 18.9, 18.7, 14.1; (+)-ESI MS *m/z*: 557.3 $[M+H]^+$, 579.5 $[M+Na]^+$.

(*R*)-*S*-Octadecanoyl-*N*-pantothenoylcysteamine acetonide

Colorless oil (198 mg, 0.34 mmol, 97%) R_f = 0.3 (EtOAc/pentane 4:1); ^{1}H NMR (400 MHz, $CDCl_3$): δ = 7.02 (br s, 1H), 6.10 (br s, 1H), 4.08 (s, 1H), 3.69 (d, *J* = 11.3 Hz, 1H), 3.61-3.51 (m, 2H), 3.50-3.39 (m, 2H), 3.28 (d, *J* = 11.5 Hz, 1H), 3.01 (t, *J* = 6.5 Hz, 2H), 2.56 (t, *J* = 7.3 Hz, 2H), 2.42 (t, *J* = 6.3 Hz, 2H), 1.69-1.60 (m, 2H), 1.46 (s, 3H), 1.42 (s, 3H), 1.32-1.24 (m, 28H), 1.04 (s, 3H), 0.97 (s, 3H), 0.88 (t, *J* = 6.8 Hz, 3H); ^{13}C NMR (100 MHz, $CDCl_3$): δ = 199.9, 171.1, 170.1, 99.1, 77.2, 71.5, 44.2, 39.6, 36.0, 34.8, 33.0, 31.9, 29.7, 29.7, 29.7, 29.7, 29.7, 29.7, 29.6, 29.6, 29.5, 29.4, 29.4, 29.2, 29.0, 28.5, 25.6, 22.7, 22.1, 18.9, 18.7, 14.1; (+)-ESI MS *m/z*: 585.3 $[M+H]^+$, 607.5 $[M+Na]^+$.

(*R*)-*S*-Icosanoyl-*N*-pantothenoylcysteamine acetonide

Colorless oil (134 mg, 0.22 mmol, 67%) R_f = 0.3 (EtOAc/pentane 2:1); ^{1}H NMR (400 MHz, $CDCl_3$): δ = 7.03 (br s, 1H), 6.17 (br s, 1H), 4.08 (s, 1H), 3.69 (d, *J* = 11.5 Hz, 1H), 3.61-3.51 (m, 2H), 3.50-3.40 (m, 2H), 3.28 (d, *J* = 11.5 Hz, 1H), 3.01 (t,

J = 6.5 Hz, 2H), 2.56 (t, *J* = 7.5 Hz, 2H), 2.42 (t, *J* = 6.1 Hz, 2H), 1.69-1.61 (m, 2H), 1.46 (s, 3H), 1.42 (s, 3H), 1.32-1.25 (m, 32H), 1.04 (s, 3H), 0.97 (s, 3H), 0.88 (t, *J* = 6.8 Hz, 3H); ^{13}C NMR (100 MHz, $CDCl_3$): δ = 199.9, 171.1, 170.1, 99.1, 77.2, 71.4, 44.2, 39.6, 39.5, 36.0, 35.9, 34.8, 33.0, 31.9, 29.7, 29.7, 29.7, 29.7, 29.6, 29.6, 29.6, 29.5, 29.4, 29.3, 29.2, 28.9, 28.4, 28.4, 25.6, 22.7, 22.1, 18.9, 18.7, 14.1; (+)-ESI MS *m/z*: 613.4 $[M+H]^+$, 635.6 $[M+Na]^+$.

(*R*)-*S*-(3-Hydroxydecanoyl)-*N*-pantothenoylcysteamine acetonide

Colorless oil (47 mg, 0.10 mmol, 76%) R_f = 0.3 (EtOAc); ^{1}H NMR (400 MHz, $CDCl_3$): δ = 7.01 (br s, 1H), 6.4 (br s, 1H), 4.13-4.08 (m, 1H), 4.08 (s, 1H), 3.69 (d, *J* = 11.5 Hz, 1H), 3.59-3.50 (m, 2H), 3.49-3.34 (m, 2H), 3.29 (d, *J* = 11.8 Hz, 1H), 3.16-3.08 (m, 1H), 3.04-2.96 (m, 1H), 2.75-2.65 (m, 2H), 2.44-2.40 (m, 2H), 1.62-1.50 (m, 2H), 1.47 (s, 3H), 1.42 (s, 3H), 1.34-1.23 (m, 10H), 1.04 (s, 3H), 0.97 (s, 3H), 0.88 (t, *J* = 6.8 Hz, 3H); ^{13}C NMR (100 MHz, $CDCl_3$): δ = 199.2, 171.1, 170.5, 99.1, 77.2, 71.4, 69.0, 51.2, 39.1, 37.1, 36.3, 35.2, 33.0, 31.8, 29.5, 29.4, 29.2, 28.7, 25.5, 22.6, 22.1, 18.9, 18.7, 14.1; (+)-ESI MS *m/z*: 489.2 $[M+H]^+$.

(*R*)-*S*-((*Z*)-7-Tetradecenoyl)-*N*-pantothenoylcysteamine acetonide

Colorless oil (251 mg, 0.48 mmol, 55%) R_f = 0.3 (EtOAc/pentane 4:1); ^{1}H NMR (400 MHz, $CDCl_3$): δ = 7.03 (br s, 1H), 6.15 (br s, 1H), 5.39-5.33 (m, 2H), 4.08 (s, 1H), 3.69 (d, *J* = 11.8 Hz, 1H), 3.61-3.50 (m, 2H), 3.48-3.40 (m, 2H), 3.28 (d, *J* = 11.8 Hz, 1H), 3.01 (t, *J* = 6.5 Hz, 2H), 2.56 (t, *J* = 7.5 Hz, 2H), 2.42 (t, *J* = 6.3 Hz, 2H), 2.04-1.94 (m, 4H), 1.71-1.64 (m, 2H), 1.46 (s, 3H), 1.42 (s, 3H), 1.35-1.24 (m, 12H), 1.04 (s, 3H), 0.97 (s, 3H), 0.88 (t, *J* = 6.5 Hz, 3H); ^{13}C NMR (100 MHz, $CDCl_3$): δ = 199.8, 171.1, 170.1, 129.8, 129.3, 99.1, 77.1, 71.4, 44.1, 39.6, 36.0, 34.8, 33.0, 32.3, 31.7, 29.7, 29.6,

29.1, 28.8, 28.6, 28.4, 27.2, 25.5, 22.6, 22.1, 18.9, 18.7, 14.1; (+)-ESI MS *m/z*: 527.1 $[M+H]^+$.

(*R*)-*S*-((*Z*)-9-Hexadecenoyl)-*N*-pantothenoylcysteamine acetonide

Colorless oil (193 mg, 0.35 mmol, 88%) R_f = 0.4 (EtOAc/pentane 4:1); ^1H NMR (400 MHz, $CDCl_3$): δ = 7.03 (br s, 1H), 6.17 (br s, 1H), 5.39-5.33 (m, 2H), 4.08 (s, 1H), 3.69 (d, *J* = 11.8 Hz, 1H), 3.61-3.49 (m, 2H), 3.48-3.38 (m, 2H), 3.28 (d, *J* = 11.8 Hz, 1H), 3.01 (t, *J* = 6.5 Hz, 2H), 2.56 (t, *J* = 7.5 Hz, 2H), 2.42 (t, *J* = 6.3 Hz, 2H), 2.05-1.94 (m, 4H), 1.75-1.61 (m, 2H), 1.46 (s, 3H), 1.42 (s, 3H), 1.34-1.24 (m, 16H), 1.04 (s, 3H), 0.97 (s, 3H), 0.88 (t, *J* = 6.8 Hz, 3H); ^{13}C NMR (100 MHz, $CDCl_3$): δ = 199.8, 171.1, 170.1, 130.0, 129.7, 99.1, 77.1, 71.4, 44.1, 39.6, 36.0, 34.8, 33.0, 31.8, 29.7, 29.7, 29.5, 29.1, 29.0, 29.0, 28.9, 28.4, 27.2, 27.1, 25.6, 22.6, 22.1, 18.9, 18.7, 14.1; (+)-ESI MS *m/z*: 555.5 $[M+H]^+$.

(*R*)-*S*-((*Z*)-9-Octadecenoyl)-*N*-pantothenoylcysteamine acetonide

Colorless oil (199 mg, 0.34 mmol, 97%) R_f = 0.3 (EtOAc/pentane 4:1); ^1H NMR (400 MHz, $CDCl_3$): δ = 7.03 (br s, 1H), 6.18 (br s, 1H), 5.37-5.31 (m, 2H), 4.08 (s, 1H), 3.69 (d, *J* = 11.5 Hz, 1H), 3.61-3.49 (m, 2H), 3.48-3.38 (m, 2H), 3.28 (d, *J* = 11.5 Hz, 1H), 3.01 (t, *J* = 6.5 Hz, 2H), 2.56 (t, *J* = 7.5 Hz, 2H), 2.43 (t, *J* = 6.3 Hz, 2H), 2.05-1.98 (m, 4H), 1.69-1.61 (m, 2H), 1.46 (s, 3H), 1.42 (s, 3H), 1.34-1.24 (m, 20H), 1.04 (s, 3H), 0.97 (s, 3H), 0.88 (t, *J* = 6.8 Hz, 3H); ^{13}C NMR (100 MHz, $CDCl_3$): δ = 199.8, 171.1, 170.1, 130.0, 129.7, 99.1, 77.1, 71.4, 44.1, 39.6, 36.0, 34.8, 33.0, 31.8, 29.7, 29.7, 29.5, 29.5, 29.3, 29.3, 29.1, 29.0, 29.0, 28.4, 27.2, 27.1, 25.6, 22.7, 22.1, 18.9, 18.7, 14.1; (+)-ESI MS *m/z*: 583.2 $[M+H]^+$.

(*R*)-*S*-((*Z*)-11-Octadecenoyl)-*N*-pantothenoylcysteamine acetonide

Colorless oil (197 mg, 0.34 mmol, 97%) R_f = 0.3 (EtOAc/pentane 4:1); ^{1}H NMR (400 MHz, $CDCl_3$): δ = 7.02 (br s, 1H), 6.10 (br s, 1H), 5.39-5.33 (m, 2H), 4.08 (s, 1H), 3.69 (d, *J* = 11.5 Hz, 1H), 3.62-3.50 (m, 2H), 3.49-3.38 (m, 2H), 3.28 (d, *J* = 11.8 Hz, 1H), 3.01 (t, *J* = 6.5 Hz, 2H), 2.56 (t, *J* = 7.5 Hz, 2H), 2.42 (t, *J* = 6.1 Hz, 2H), 2.05-1.94 (m, 4H), 1.68-1.61 (m, 2H), 1.46 (s, 3H), 1.42 (s, 3H), 1.35-1.24 (m, 20H), 1.04 (s, 3H), 0.97 (s, 3H), 0.88 (t, *J* = 6.8 Hz, 3H); ^{13}C NMR (100 MHz, $CDCl_3$): δ = 199.8, 171.1, 170.1, 130.0, 129.8, 99.1, 77.2, 71.5, 44.2, 39.6, 36.0, 34.8, 33.0, 31.8, 29.7, 29.6, 29.5, 29.5, 29.4, 29.2, 29.0, 29.0, 28.8, 28.5, 27.2, 27.2, 25.6, 22.7, 22.1, 18.9, 18.7, 14.1; (+)-ESI MS *m/z*: 583.5 $[M+H]^+$.

(*R*)-*S*-((2*E*,11*Z*)-2,11-Octadecadienoyl)-*N*-pantothenoylcysteamine acetonide

Colorless oil (118 mg, 0.20 mmol, 67%) R_f = 0.3 (EtOAc/pentane 2:1); ^{1}H NMR (400 MHz, $CDCl_3$): δ = 7.04 (br s, 1H), 6.93 (dt, *J* = 15.6, 7.0 Hz, 1H), 6.13 (br s, 1H), 6.1 (t, *J* = 1.5 Hz, 1H), 5.39-5.31 (m, 2H), 4.08 (s, 1H), 3.69 (d, *J* = 11.8 Hz, 1H), 3.62-3.51 (m, 2H), 3.49-3.41 (m, 2H), 3.28 (d, *J* = 11.5 Hz, 1H), 3.08 (t, *J* = 6.4 Hz, 2H), 2.42 (t, *J* = 6.1 Hz, 2H), 2.23-2.17 (m, 2H), 2.06-1.99 (m, 4H), 1.46 (s, 3H), 1.42 (s, 3H), 1.40-1.23 (m, 18H), 1.04 (s, 3H), 0.97 (s, 3H), 0.88 (t, *J* = 6.8 Hz, 3H); ^{13}C NMR (100 MHz, $CDCl_3$): δ = 190.1, 171.2, 170.1, 146.8, 130.0, 129.7, 128.2, 99.1, 77.1, 71.4, 39.7, 35.9, 34.8, 33.0, 32.2, 31.8, 29.7, 29.7, 29.5, 29.2, 29.1, 29.1, 29.0, 28.2, 27.9, 27.2, 27.1, 22.7, 22.1, 18.9, 18.7, 14.1; (+)-ESI MS *m/z*: 581.0 $[M+H]^+$.

(*R*)-*S*-Acyl-*N*-pantothenoylcysteamines (10–13)

The thioesters **9** were dissolved in AcOH/H_2O 2:1 (2 mL) and stirred for 5 h at room temperature. The solvent was evaporated in vacuo and the crude product was used for incubation experiments without further purification [13].

(*R*)-*S*-Butanoyl-*N*-pantothenoylcysteamine (10a)

Crude product (18.9 mg, 0.05 mmol, 99%); ^{1}H NMR (500 MHz, CD_3OD): δ = 3.88 (s, 1H), 3.51-3.33 (m, 6H), 3.00 (t, *J* = 6.7 Hz, 2H), 2.56 (t, *J* = 7.5 Hz, 2H), 2.40 (t, *J* = 6.7 Hz, 2H), 1.67 (sxt, *J* = 7.4 Hz, 2H), 0.95 (t, *J* = 7.3 Hz, 3H), 0.91 (s, 6H); ^{13}C NMR (125 MHz, CD_3OD): δ = 200.6, 176.1, 173.9, 77.3, 70.4, 46.7, 40.4, 40.1, 36.4, 36.3, 29.1, 21.3, 20.9, 20.2, 13.8; HRMS (ESI +) *m/z*: 371.1612 $[M+Na]^+$.

(*R*)-*S*-Hexanoyl-*N*-pantothenoylcysteamine (10b)

Crude product (15.4 mg, 0.04 mmol, 99%); ^{1}H NMR (400 MHz, CD_3OD): δ = 3.88 (s, 1H), 3.53-3.33 m, 6H), 2.99 (t, *J* = 6.8 Hz, 2H), 2.57 (t, *J* = 7.4 Hz, 2H), 2.40 (t, *J* = 6.7 Hz, 2H), 1.65 (quin, *J* = 7.2 Hz, 2H), 1.36-1.29 (m, 4H), 0.91, (s, 6H), 0.91 (t, *J* = 7.02 Hz, 3H); ^{13}C NMR (100 MHz, CD_3OD): δ = 200.7, 176.1, 173.9, 77.3, 70.4, 44.8, 40.4, 40.1, 36.4, 36.4, 32.2, 29.1, 26.4, 23.4, 21.4, 21.0, 14.2; HRMS (ESI +) *m/z*: 399.1922 $[M+Na]^+$.

(*R*)-*S*-Octanoyl-*N*-pantothenoylcysteamine (10c)

Crude product (7.9 mg, 0.01 mmol, 99%); ^{1}H NMR (500 MHz, CD_3OD): δ = 3.88 (s, 1H), 3.52-3.33 (m, 6H), 2.99 (t, *J* = 6.7 Hz, 2H), 2.57 (t, *J* = 7.5 Hz, 2H), 2.40 (t, *J* = 6.7 Hz, 2H), 1.64 (quin, *J* = 7.3 Hz, 2H), 1.35-1.25 (m, 8H), 0.91 (s, 6H), 0.90 (t, *J* = 7.02 Hz, 3H); ^{13}C NMR (125 MHz, CD_3OD): δ = 200.7, 176.1, 173.9, 77.3, 70.4,

44.9, 40.4, 40.1, 36.4, 36.4, 32.8, 30.1, 30.0, 29.1, 26.7, 23.7, 21.4, 20.9, 14.4; HRMS (ESI +) *m/z*: 427.2239 $[M+Na]^+$.

(*R*)-*S*-Decanoyl-*N*-pantothenoylcysteamine (10d)

Crude product (12.7 mg, 0.02 mmol, 99%); ^{1}H NMR (500 MHz, CD_3OD): δ= 3.88 (s, 1H), 3.52-3.37 (m, 6H), 2.99 (t, *J* = 6.7 Hz, 2H), 2.57 (t, *J* = 7.5 Hz, 2H), 2.40 (t, *J* = 6.7 Hz, 2H), 1.64 (quin, *J* = 7.2 Hz, 2H), 1.35-1.22 (m, 12H), 0.91 (s, 6H), 0.90 (t, *J* = 7.02 Hz, 3H); ^{13}C NMR (125 MHz, CD_3OD): δ= 200.7, 176.1, 173.9, 77.3, 70.4, 44.9, 40.4, 40.2, 36.4, 36.4, 33.1, 30.5, 30.4, 30.4, 30.0, 29.1, 26.7, 23.8, 21.4, 21.0, 14.5; HRMS (ESI +) *m/z*: 433.2733 $[M+H]^+$, 455.2554 $[M+Na]^+$.

(*R*)-*S*-Dodecanoyl-*N*-pantothenoylcysteamine (10e)

Crude product (16 mg, 0.03 mmol, 99%); ^{1}H NMR (500 MHz, CD_3OD): δ= 3.88 (s, 1H), 3.52-3.37 (m, 6H), 2.99 (t, *J* = 6.7 Hz, 2H), 2.57 (t, *J* = 7.3 Hz, 2H), 2.40 (t, *J* = 6.7 Hz, 2H), 1.64 (quin, *J* = 7.2 Hz, 2H), 1.34-1.27 (m, 16H), 0.91 (s, 6H), 0.90 (t, *J* = 7.0 Hz, 3H); ^{13}C NMR (125 MHz, CD_3OD): δ= 200.7, 176.1, 173.9, 77.3, 70.4, 44.9, 40.4, 40.2, 36.4, 36.4, 33.1, 30.7, 30.7, 30.6, 30.5, 30.4, 30.0, 29.2, 26.7, 23.8, 21.4, 21.0, 14.5; HRMS (ESI +) *m/z*: 483.2868 $[M+Na]^+$.

(*R*)-*S*-Tetradecanoyl-*N*-pantothenoylcysteamine (10f)

Crude product (6.1 mg, 0.01 mmol, 98%); ^{1}H NMR (500 MHz, CD_3OD): δ= 3.88 (s, 1H), 3.52-3.33 (m, 6H), 2.99 (t, *J* = 6.7 Hz, 2H), 2.57 (t, *J* = 7.3 Hz, 2H), 2.40 (t, *J* = 6.7 Hz, 2H), 1.64 (quin, *J* = 7.2 Hz, 2H), 1.36-1.26 (m, 20H), 0.91 (s, 6H), 0.90 (t, *J* = 7.3 Hz, 3H); ^{13}C NMR (125 MHz, CD_3OD): δ= 200.7, 176.1, 173.9, 77.3, 70.4, 44.9,

40.4, 40.1, 36.4, 36.4, 33.1, 30.8, 30.8, 30.8, 30.7, 30.6, 30.5, 30.4, 30.0, 29.1, 26.7, 23.8, 21.4, 20.9, 14.5; HRMS (ESI +) *m/z*: 511.3175 $[M+Na]^+$.

(*R*)-*S*-Hexadecanoyl-*N*-pantothenoylcysteamine (10g)

Crude product (5.8 mg, 0.01 mmol, 99%); ^{1}H NMR (500 MHz, CD_3OD): δ= 3.88 (s, 1H), 3.52-3.3.33 (m, 6H), 2.99 (t, *J* = 6.7 Hz, 2H), 2.57 (t, *J* = 7.3 Hz, 2H), 2.40 (t, *J* = 6.7 Hz, 2H), 1.64 (quin, *J* = 7.2 Hz, 2H), 1.33-1.25 (m, 24H), 0.91 (s, 6H), 0.90 (t, *J* = 7.0 Hz, 3H); ^{13}C NMR (125 MHz, CD_3OD): δ= 200.7, 176.1, 173.9, 77.3, 70.4, 44.9, 40.4, 40.1, 36.4, 36.4, 33.1, 30.8, 30.8, 30.8, 30.8, 30.8, 30.7, 30.6, 30.5, 30.4, 30.0, 29.1, 26.7, 23.8, 21.4, 21.0, 14.5; HRMS (ESI +) *m/z*: 539.3489 $[M+Na]^+$.

(*R*)-*S*-Octadecanoyl-*N*-pantothenoylcysteamine (10h)

Crude product (3.4 mg, 0.006 mmol, 99%); ^{1}H NMR (600 MHz, CD_3OD): δ= 3.88 (s, 1H), 3.51-3.32 (m, 6H), 2.99 (t, *J* = 6.8 Hz, 2H), 2.57 (t, *J* = 7.4 Hz, 2H), 2.40 (t, *J* = 6.7 Hz, 2H), 1.64 (quin, *J* = 7.3 Hz, 2H), 1.33-1.28 (m, 28H), 0.91 (s, 6H), 0.90 (t, *J* = 7.1 Hz, 3H); ^{13}C NMR (150 MHz, CD_3OD): δ= 200.7, 176.1, 173.9, 77.3, 70.4, 44.9, 40.4, 40.1, 36.4, 36.4, 33.1, 30.8, 30.8, 30.8, 30.8, 30.8, 30.8, 30.8, 30.7, 30.6, 30.5, 30.4, 30.0, 29.1, 26.7, 23.8, 21.4, 20.9, 14.5; HRMS (ESI +) *m/z*: 567.3802 $[M+Na]^+$.

(*R*)-*S*-Icosanoyl-*N*-pantothenoylcysteamine (10i)

Crude product (21.2 mg, 0.03 mmol, 98%); ^{1}H NMR (600 MHz, CD_3OD): δ= 3.88 (s, 1H), 3.51-3.33 (m, 6H), 2.99 (t, *J* = 6.1 Hz, 2H), 2.57 (t, *J* = 7.0 Hz, 2H), 2.39 (t, *J* = 6.7 Hz, 2H), 1.64 (quin, *J* = 7.2 Hz, 2H), 1.34-1.28 (m, 32H), 0.91 (s, 6H), 0.89 (t, *J* = 7.1 Hz, 3H); ^{13}C NMR (150 MHz, CD_3OD): δ= 200.5, 176.1, 173.9, 77.3, 70.4, 44.9, 44.4, 40.2, 36.4, 36.3, 34.0, 33.1, 30.8, 30.8, 30.8, 30.8, 30.8, 30.8, 30.8, 30.7, 30.6,

30.5, 30.4, 30.0, 29.8, 29.2, 26.7, 23.8, 21.4, 21.0, 14.5; HRMS (ESI +) *m/z*: 595.4117 $[M+Na]^+$.

(*R*)-*S*-(3-Hydroxydecanoyl)-*N*-pantothenoylcysteamine (12)

Crude product (12.8 mg, 0.02 mmol, 99%); ^{1}H NMR (500 MHz, CD_3OD): δ= 4.04-3.99 (m, 1H), 3.89 (s, 1H), 3.52-3.32 (m, 6H), 3.02 (t, *J* = 6.7 Hz, 2H), 2.73-2.65 (m, 2H), 2.40 (t, *J* = 6.6 Hz, 2H), 1.47-1.44 (m, 2H), 1.36-1.28 (m, 10H), 0.92 (s, 6H), 0.90 (t, *J* = 7.0 Hz, 3H); ^{13}C NMR (125 MHz, CD_3OD): δ= 198.9, 176.1, 174.0, 77.3, 70.4, 69.6, 52.7, 40.4, 40.0, 38.2, 36.4, 36.4, 33.0, 30.6, 30.4, 29.3, 26.6, 23.7, 21.3, 21.0, 14.5; HRMS (ESI +) *m/z*: 471.2505 $[M+Na]^+$.

(*R*)-*S*-((*Z*)-7-Tetradecenoyl)-*N*-pantothenoylcysteamine (11a)

Crude product (15.6 mg, 0.02 mmol, 99%); ^{1}H NMR (500 MHz, CD_3OD): δ= 5.42-5.32 (m, 2H), 3.89 (s, 1H), 3.52-3.33 (m, 6H), 2.99 (t, *J* = 6.7 Hz, 2H), 2.59-2.55 (m, 2H), 2.40 (t, *J* = 6.7 Hz, 2H), 1.67-1.62 (m, 4H), 1.37-1.29 (m, 14H), 0.91 (s, 6H), 0.90 (t, *J* = 7.02 Hz, 3H); ^{13}C NMR (125 MHz, CD_3OD): δ= 200.6, 176.1, 173.9, 131.8, 131.2, 77.3, 70.4, 44.8, 40.4, 40.1, 36.4, 36.4, 33.0, 30.8, 30.4, 29.9, 29.4, 29.1, 28.2, 27.9, 26.6, 23.7, 21.3, 21.0, 14.5; HRMS (ESI +) *m/z*: 509.3019 $[M+Na]^+$.

(*R*)-*S*-((*Z*)-9-Hexadecenoyl)-*N*-pantothenoylcysteamine (11b)

Crude product (191.6 mg, 0.35 mmol, 98 %); ^{1}H NMR (400 MHz, CD_3OD): δ = 5.34 (t, *J* = 4.9 Hz, 2H), 3.88 (s, 1H), 3.51-3.33 (m, 6H), 2.99 (t, *J* = 6.8 Hz, 2H), 2.57 (t, *J* = 7.3 Hz, 2H), 2.40 (t, *J* = 6.7 Hz, 2H), 2.05-1.97 (m, 4H), 1.64 (quin, *J* = 6.8 Hz, 2H), 1.38-1.27 (m, 16 H), 0.91 (s, 6H), 0.90 (t, *J* = 7.3 Hz, 3H); ^{13}C NMR (100 MHz, CD_3OD): δ = 200.6, 176.1, 173.9, 131.5, 130.8, 77.3, 70.4, 44.8, 40.4, 40.1, 36.4,

36.4, 33.0, 30.9, 30.8, 30.2, 30.1, 30.1, 30.0, 29.1, 28.2, 28.1, 26.7, 23.7, 21.4, 21.0, 14.5; HRMS (ESI +) *m/z*: 537.3334 $[M+Na]^+$.

(*R*)-*S*-((*Z*)-9-Octadecenoyl)-*N*-pantothenoylcysteamine (11c)

Crude product (16.2 mg, 0.03 mmol, 99%); ^{1}H NMR (400 MHz, CD_3OD): δ = 5.34 (t, *J* = 4.6 Hz, 2H), 3.88 (s, 1H), 3.53-3.33 (m, 6H), 2.99 (t, *J* = 6.7 Hz, 2H), 2.57 (t, *J* = 7.3 Hz, 2H), 2.40 (t, *J* = 6.5 Hz, 2H), 2.12-1.97 (m, 4H), 1.64 (quin, J = 6.8 Hz, 2H), 1.43-1.22 (m, 20H), 0.91 (s, 6H), 0.90 (t, *J* = 7.0 Hz, 3H); ^{13}C NMR (100 MHz, CD_3OD): δ = 200.6, 176.1, 173.9, 130.9, 130.8, 77.3, 70.4, 44.9, 40.4, 40.2, 36.5, 36.4, 33.1, 30.9, 30.8, 30.6, 30.5, 30.4, 30.3, 30.1, 30.0, 29.2, 28.2, 28.1, 26.7, 23.8, 21.4, 21.0, 14.5; HRMS (ESI +) *m/z*: 565.3652 $[M+Na]^+$.

(*R*)-*S*-((*Z*)-11-Octadecenoyl)-*N*-pantothenoylcysteamine (11d)

Crude product (4.4 mg, 0.008 mmol, 99%); ^{1}H NMR (600 MHz, CD_3OD): δ= 5.34 (t, *J* = 4.9 Hz, 2H), 3.88 (s, 1H), 3.50-3.33 (m, 6H), 2.99 (t, *J* = 6.7 Hz, 2H), 2.57 (t, *J* = 7.4 Hz, 2H), 2.40 (t, *J* = 6.7 Hz, 2H), 2.09-1.96 (m, 4H), 1.64 (quin, *J* = 7.1 Hz, 2H), 1.45-1.29 (m, 20H), 0.91 (s, 6H), 0.90 (t, *J* = 7.2 Hz, 3H); ^{13}C NMR (150 MHz, CD_3OD): δ= 200.7, 176.1, 173.9, 130.9, 130.8, 77.3, 70.4, 44.9, 40.4, 40.2, 36.4, 36.4, 33.1, 30.9, 30.8, 30.6, 30.5, 30.4, 30.3, 30.1, 30.0, 29.9, 29.1, 28.1, 26.7, 23.8, 21.4, 20.9, 14.5; HRMS (ESI +) *m/z*: 565.3647 $[M+Na]^+$.

(*R*)-*S*-((2*E*,11*Z*)-2,11-Octadecadienoyl)-*N*-pantothenoylcysteamine (13)

Crude product (72.6 mg, 0.13 mmol, 93%); ^{1}H NMR (400 MHz, CD_3OD): δ = 6.92 (dt, *J* = 15.3, 7.0 Hz, 1H), 6.16 (d, *J* = 15.3 Hz, 1H), 5.34 (t, *J* = 4.9 Hz, 2H), 3.88 (s, 1H), 3.51-3.33 (m, 6H), 3.06 (t, *J* = 6.8 Hz, 2H), 2.40 (t, *J* = 6.7 Hz, 2H), 2.22 (q, *J* = 7.1 Hz, 2H), 2.07-2.01 (m, 4H), 1.49-1.29 (m, 18H), 0.91 (s, 6H), 0.90 (t, *J* = 6.8 Hz,

3H); ^{13}C NMR (100 MHz, CD_3OD): δ = 191.1, 175.3, 173.9, 147.6, 130.9, 130.8, 129.6, 77.3, 70.4, 40.4, 40.2, 36.4, 36.4, 33.2, 33.0, 30.9, 30.8, 30.4, 30.3, 30.2, 30.1, 29.2, 28.9, 28.2, 28.1, 23.8, 21.4, 21.0, 14.5; HRMS (ESI +) *m/z*: 541.3671 $[M+H]^+$, 563.3491 $[M+Na]^+$.

References

1. Neumann, A.; Patzelt, D.; Wagner-Döbler, I.; Schulz, S. *ChemBioChem* **2013,** *14,* 2355–2361.
2. Müller, K.-D.; Husmann, H.; Nalik, H. P.; Schomburg, G. *Chromatographia* **1990,** *30,* 245–248.
3. Wagner-Döbler, I.; Thiel, V.; Eberl, L.; Allgaier, M.; Bodor, A.; Meyer, S.; Ebner, S.; Hennig, A.; Pukall, R.; Schulz, S. *ChemBioChem* **2005,** *6,* 2195–2206.
4. Thiel, V.; Kunze, B.; Verma, P.; Wagner-Döbler, I.; Schulz, S. *ChemBioChem* **2009,** *10,* 1861–1868.
5. Neumann, B.; Pospiech, A.; Schairer, H. U. *Trends Genet.* **1992,** *8*, 332–333.
6. Dickschat, J. S.; Pahirulzaman, K. A. K.; Rabe, P.; Klapschinski, T. A. *ChemBioChem* **2014,** *15*, 810–814.
7. Gietz, R. D.; Schiestl, R. H. *Nat. Protoc.* **2007,** *2*, nprot.2007.13.
8. Angle, S. R.; Henry, R. M. *J. Org. Chem.* **1998,** *63*, 7490–7497.
9. Patzelt, D.; Wang, H.; Buchholz, I.; Rohde, M.; Gröbe, L.; Pradella, S.; Neumann, A.; Schulz, S.; Heyber, S.; Münch, K.; Münch, R.; Jahn, D.; Wagner-Döbler, I.; Tomasch, J. *ISME J.* **2013,** *7,* 2274–2286.
10. Berger, M.; Neumann, A.; Schulz, S.; Simon, M.; Brinkhoff, T. *J. Bacteriol.* **2011,** *193,* 6576–6585.
11. Gaudelli, N. M.; Townsend, C. A. *J. Org. Chem.* **2013,** *78,* 6412–6426.
12. Neises, B.; Steglich, W. *Angew. Chem., Int. Ed. Engl.* **1978,** *17,* 522–524

13. Agarwal, V.; Diethelm, S.; Ray, L.; Garg, N.; Awakawa, T.; Dorrestein, P. C.; Moore, B. S. *Org. Lett.* **2015,** *17,* 4452–4455.

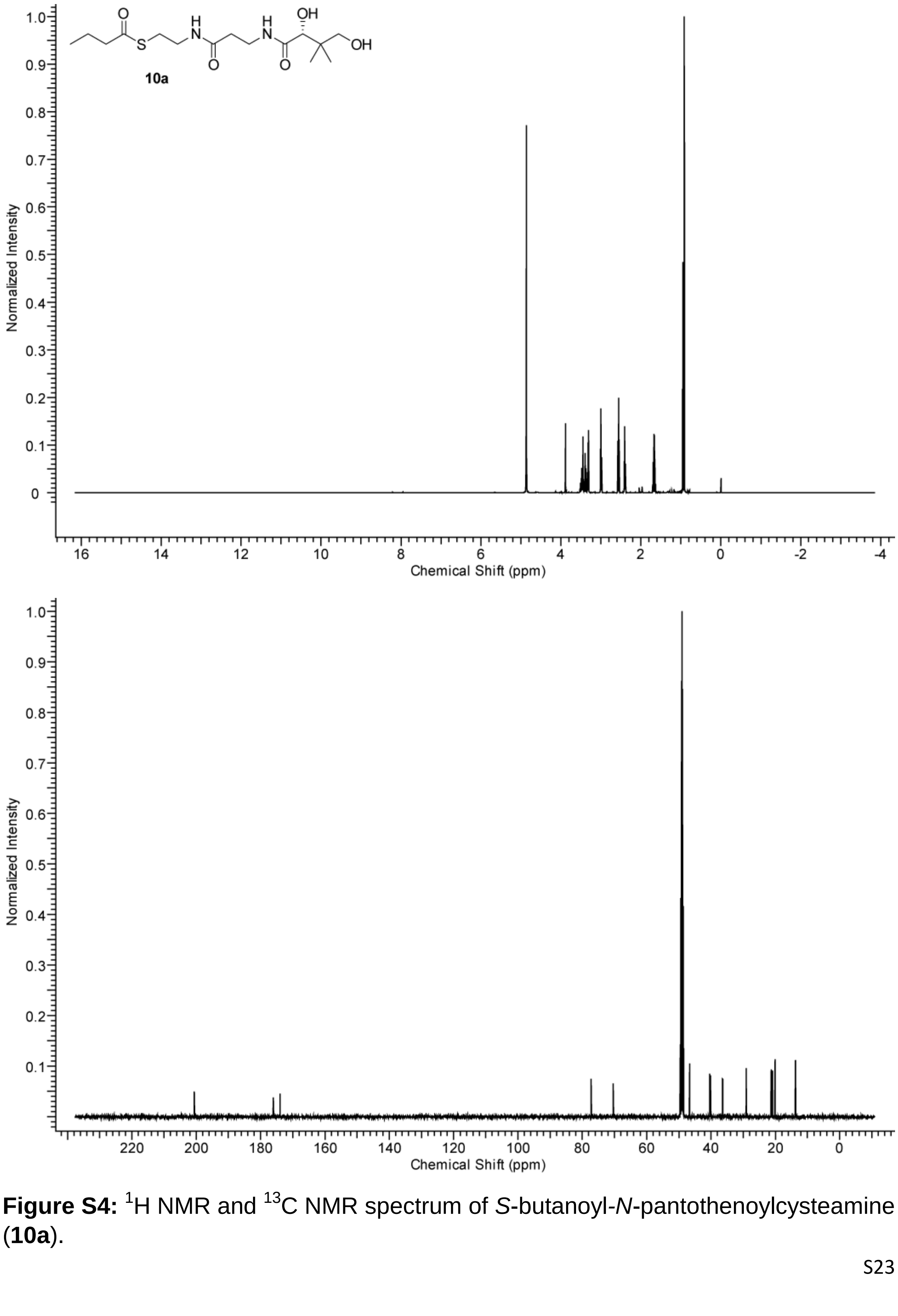

Figure S4: ^{1}H NMR and ^{13}C NMR spectrum of *S*-butanoyl-*N*-pantothenoylcysteamine (**10a**).

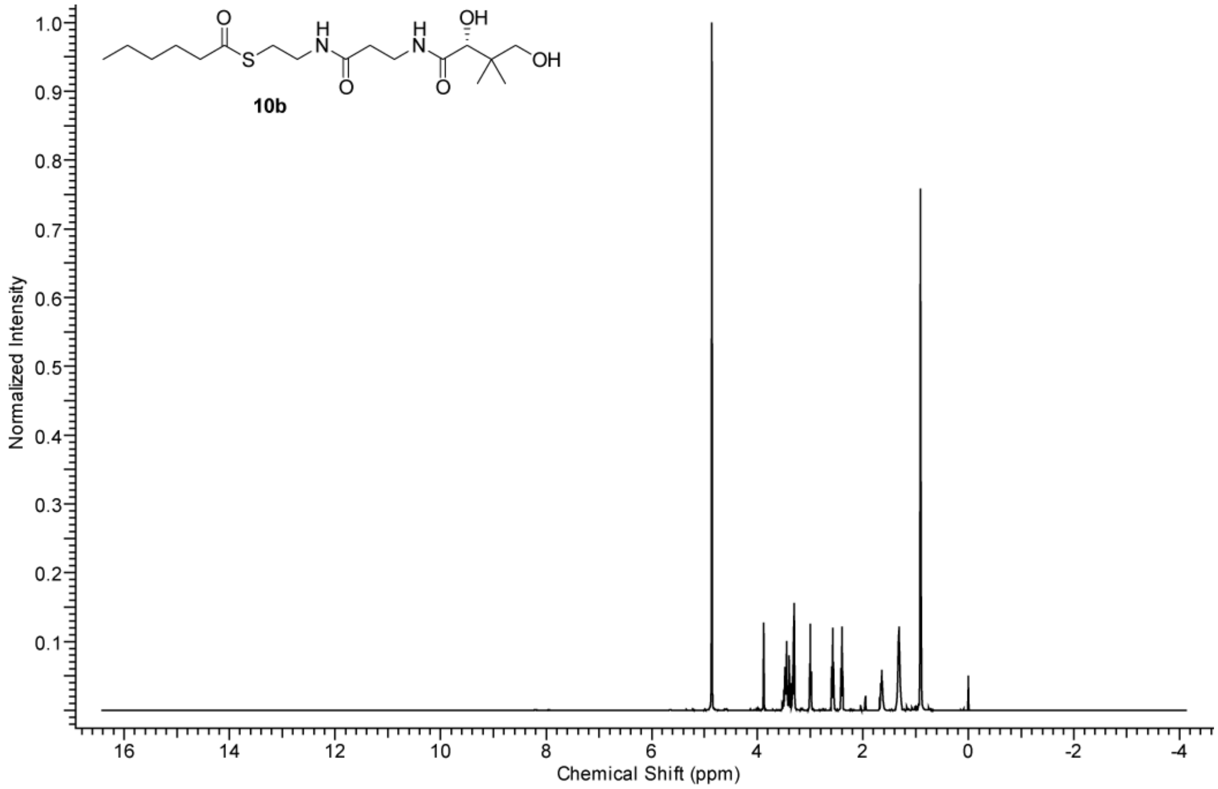

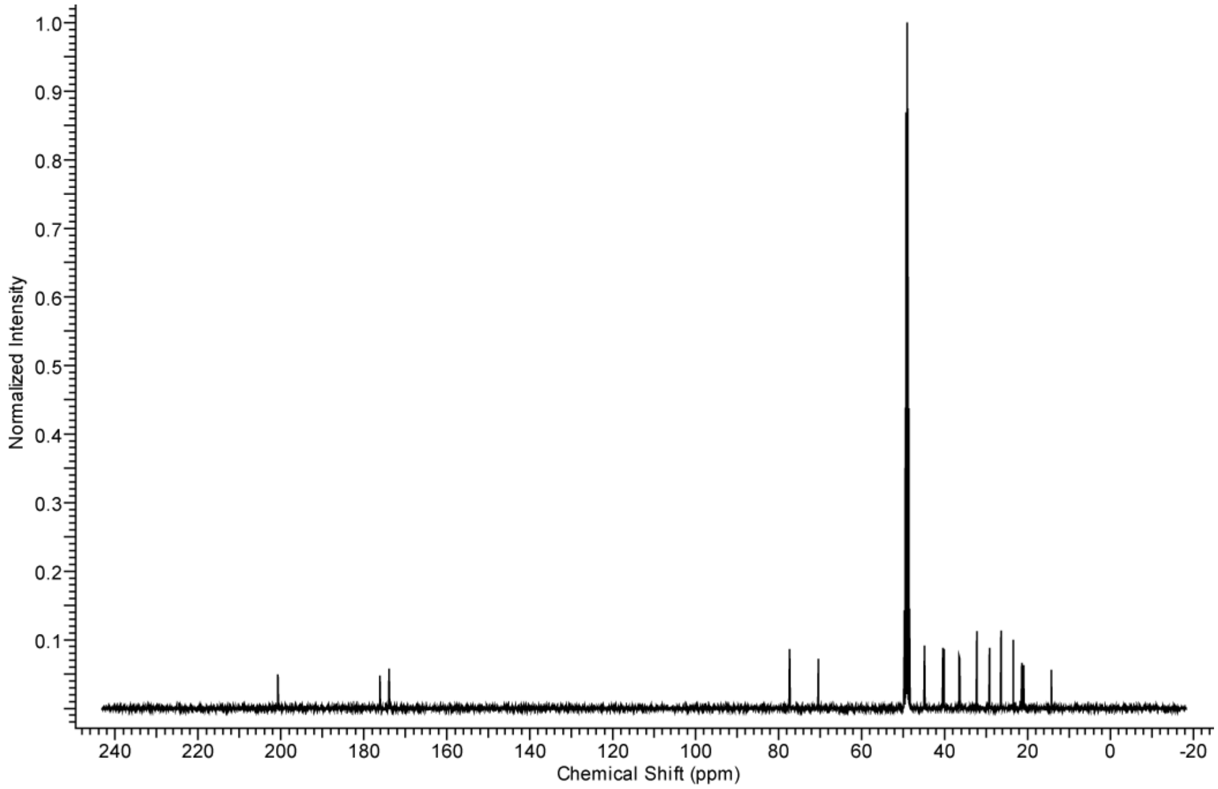

Figure S5: ^{1}H NMR and ^{13}C NMR spectrum of *S*-hexanoyl-*N*-pantothenoylcysteamine (**10b**).

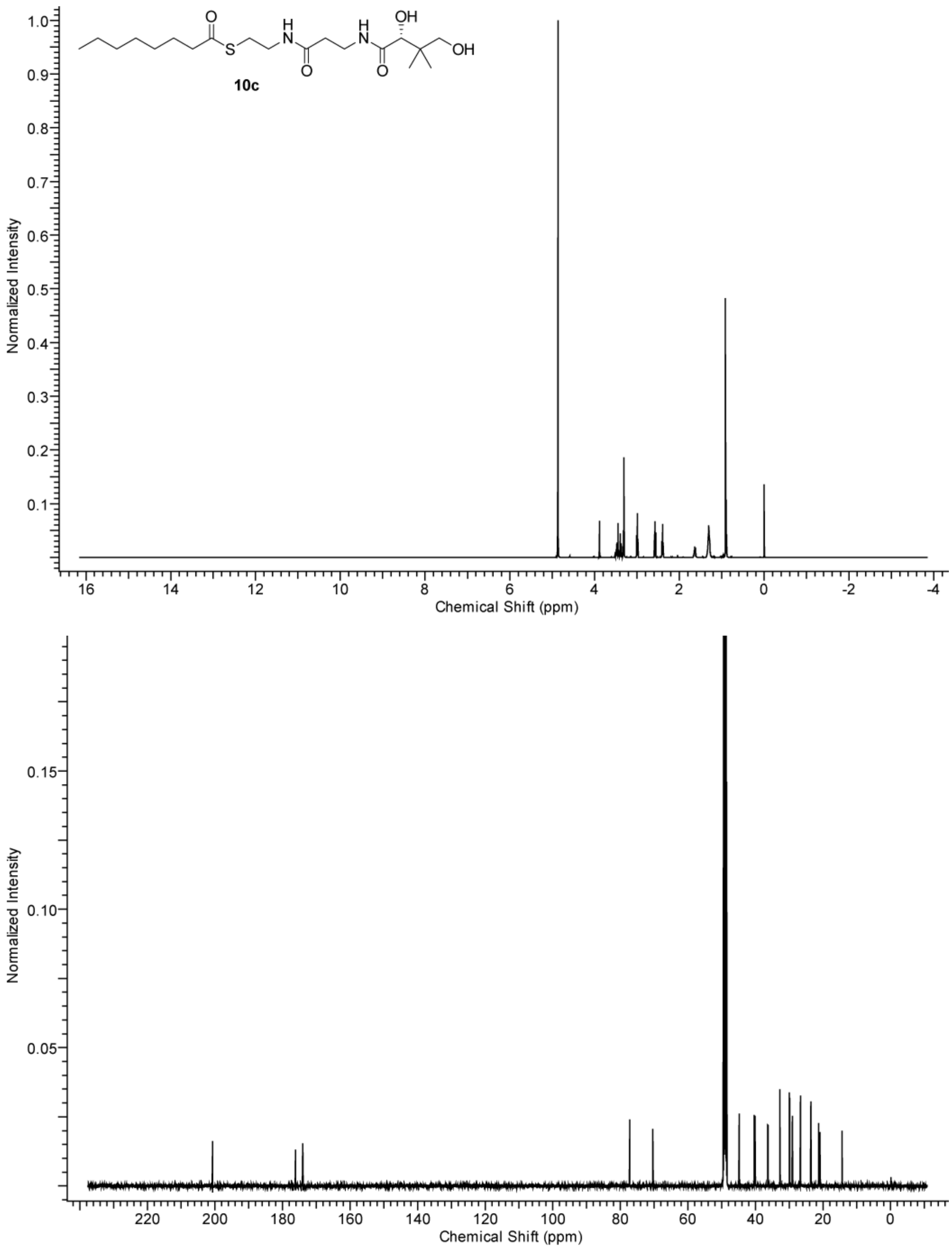

Figure S6: ^{1}H NMR and ^{13}C NMR spectrum of *S*-octanoyl-*N*-pantothenoylcysteamine (**10c**).

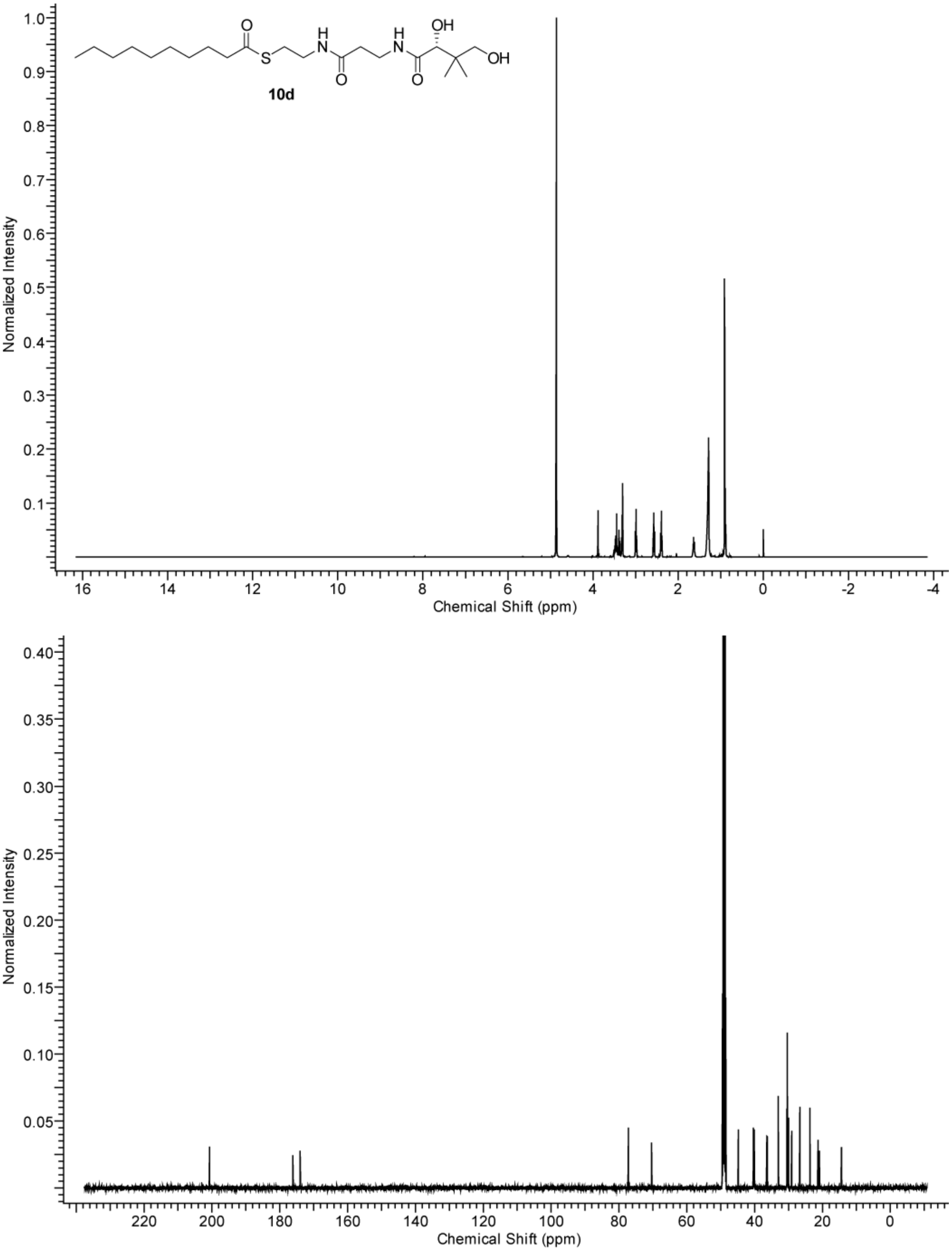

Figure S7: ^{1}H NMR and ^{13}C NMR spectrum of *S*-decanoyl-*N*-pantothenoylcysteamine (**10d**).

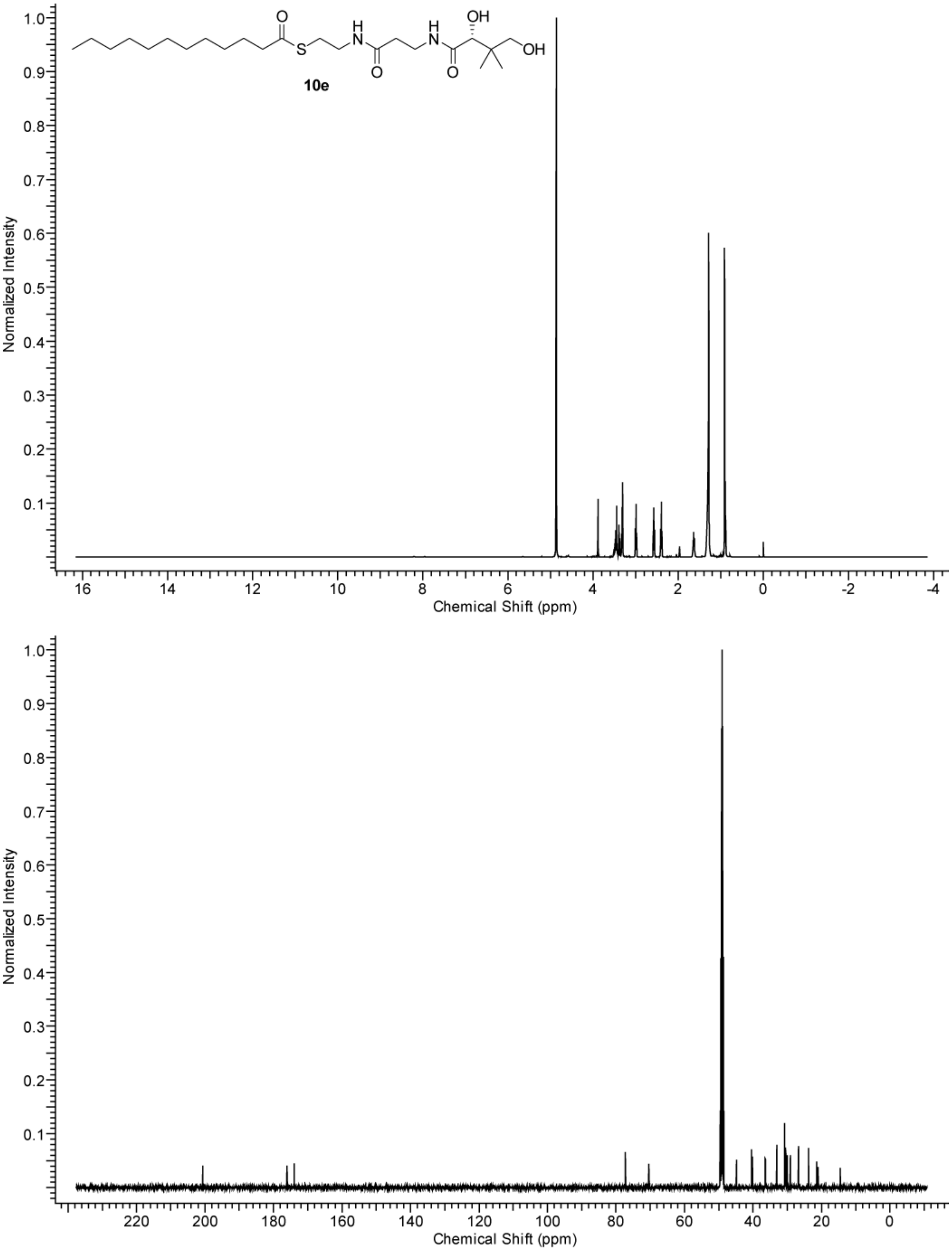

Figure S8: ^{1}H NMR and ^{13}C NMR spectrum of *S*-dodecanoyl-*N*-pantothenoylcysteamine (**10e**).

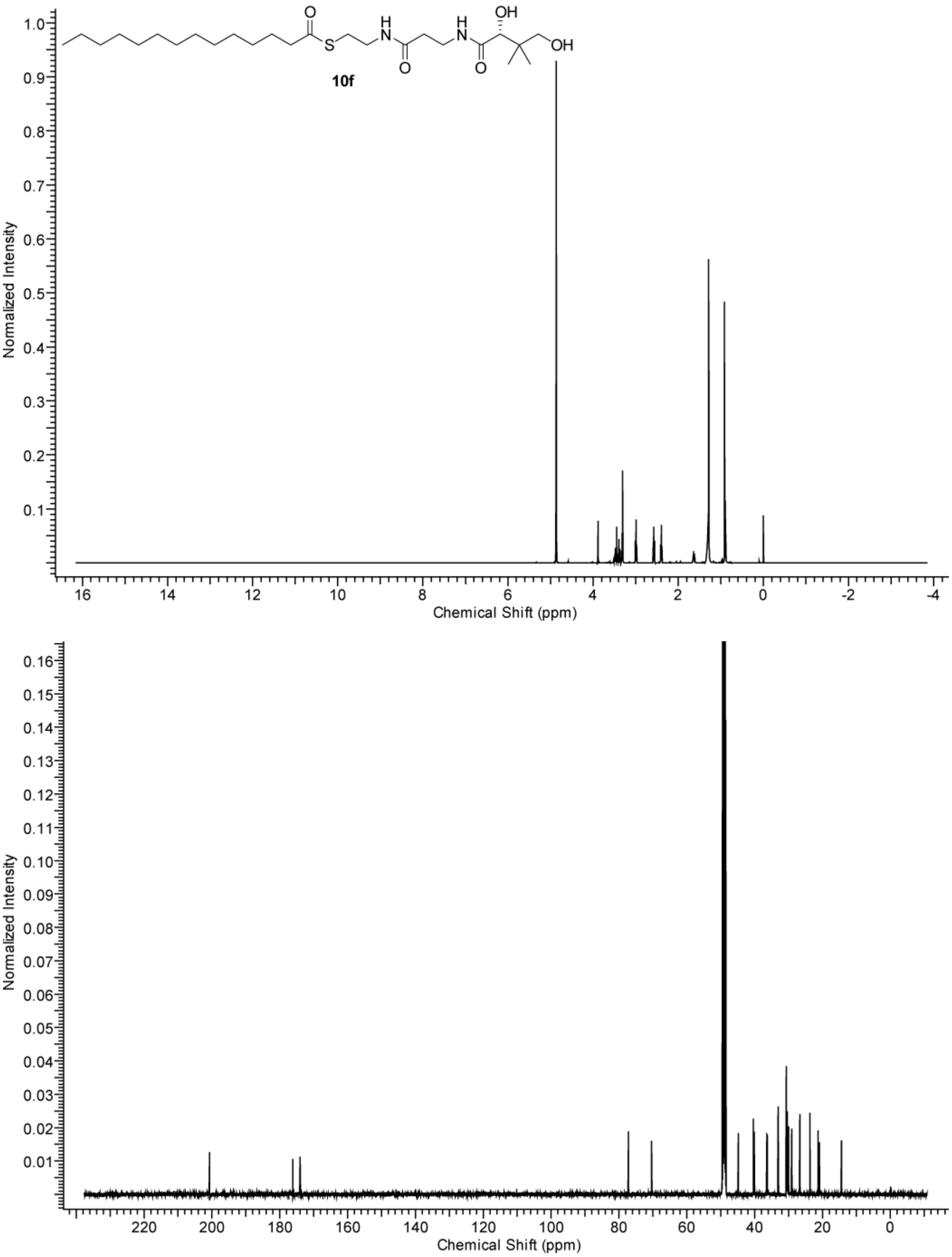

Figure S9: ^{1}H NMR and ^{13}C NMR spectrum of *S*-tetradecanoyl-*N*-pantothenoylcysteamine (**10f**).

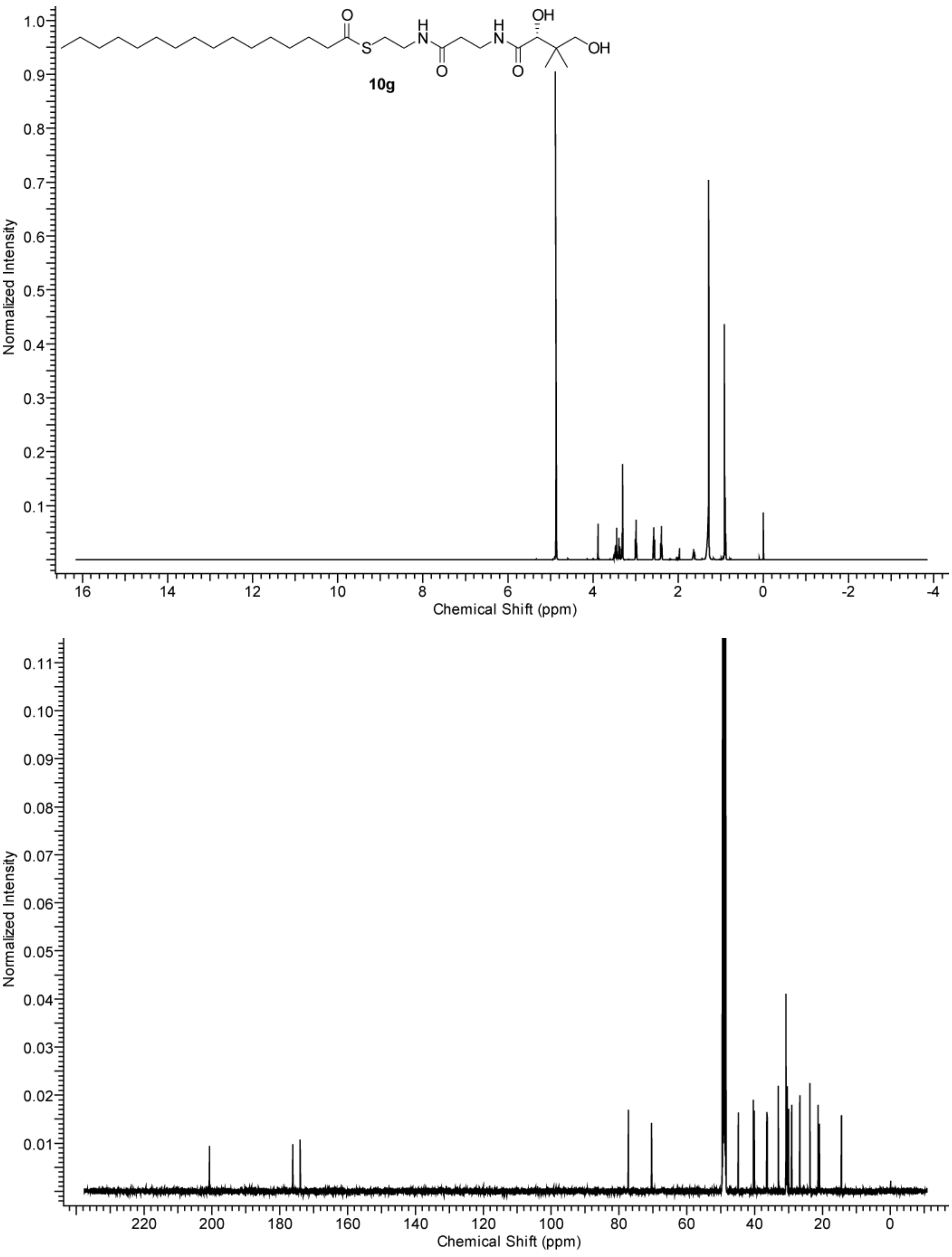

Figure S10: ^{1}H NMR and ^{13}C NMR spectrum of *S*-hexadecanoyl-*N*-pantothenoylcysteamine (**10g**).

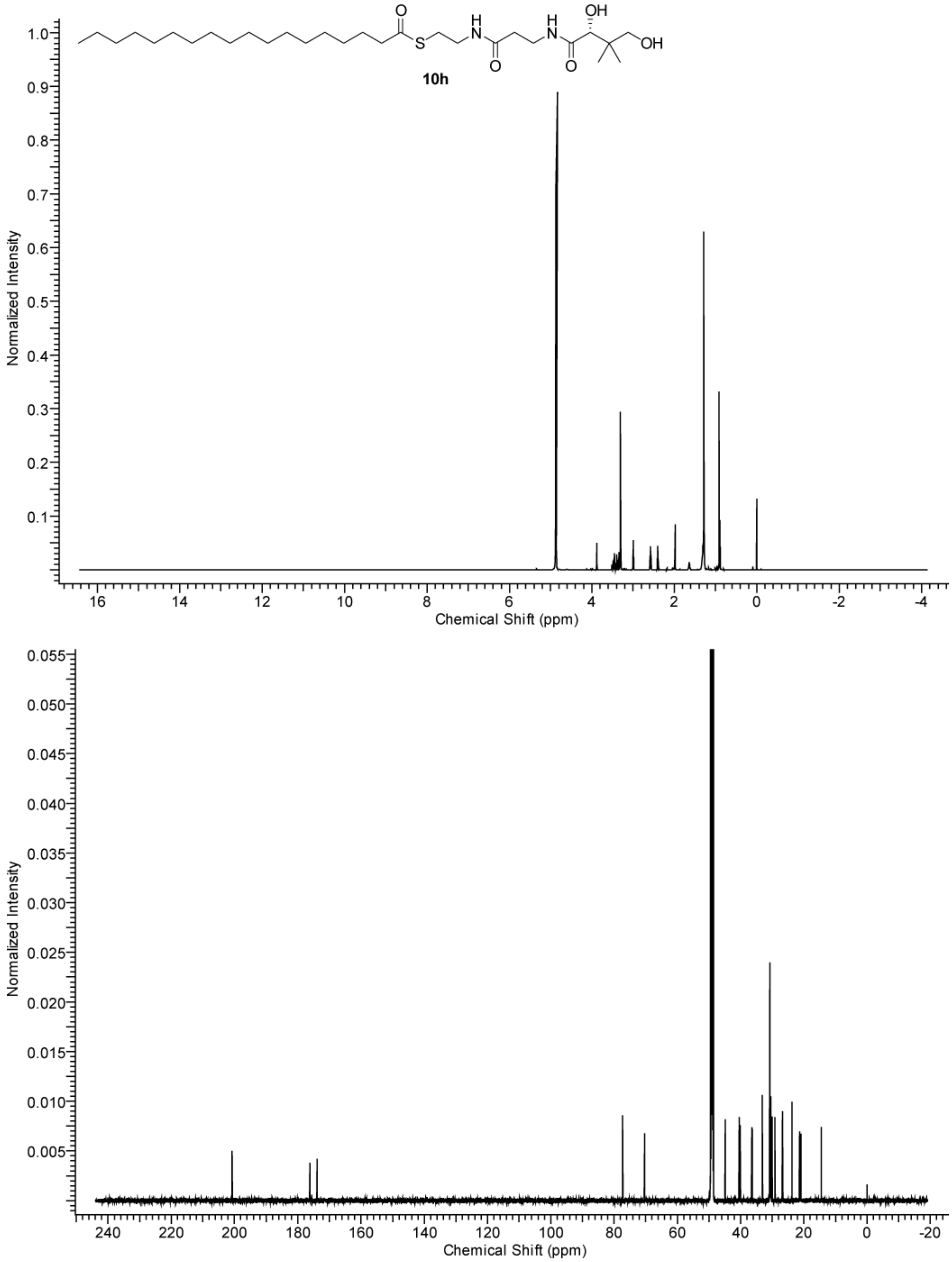

Figure S11: ^{1}H NMR and ^{13}C NMR spectrum of *S*-octadecanoyl-*N*-pantothenoylcysteamine (**10h**).

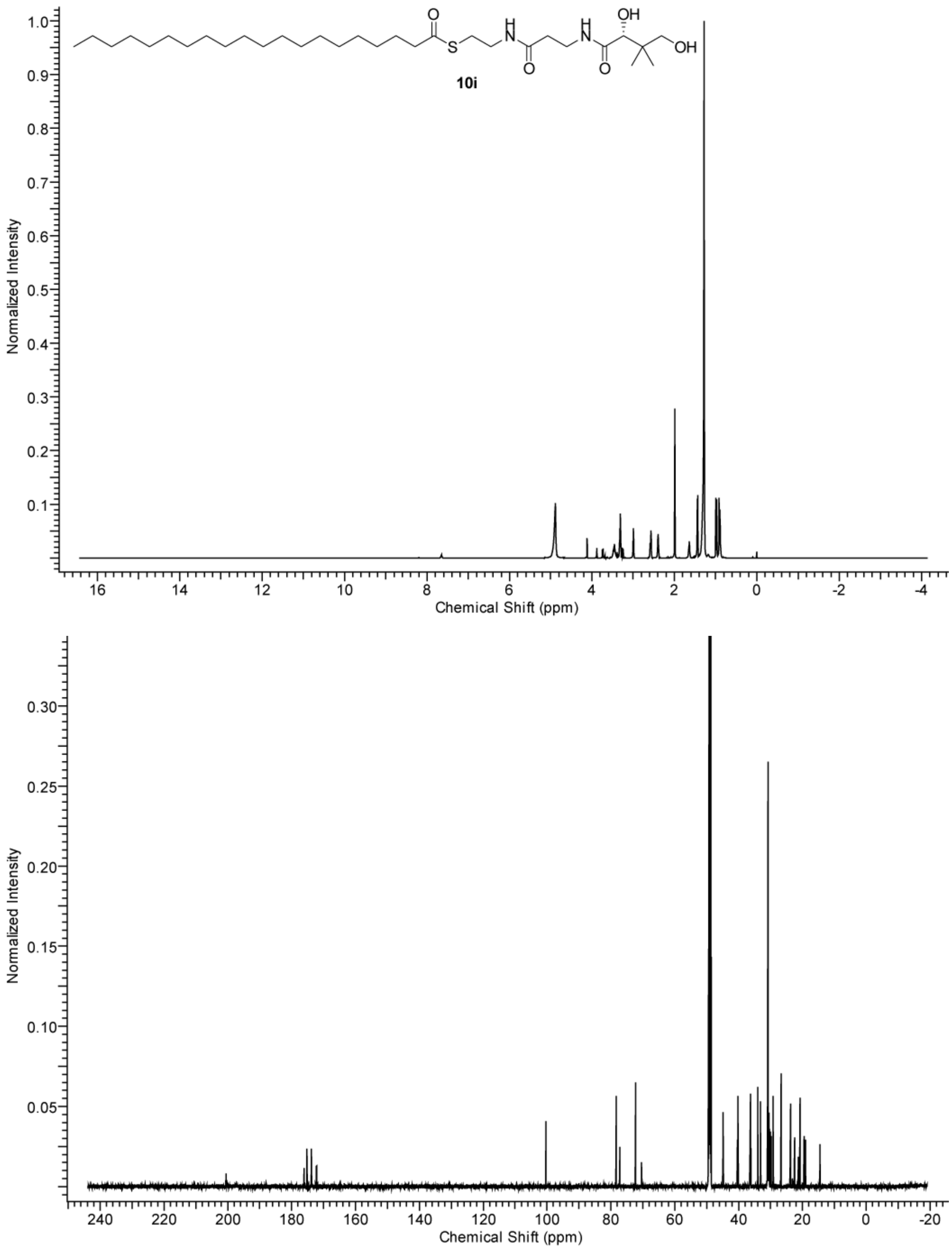

Figure S12: ^{1}H NMR and ^{13}C NMR spectrum of *S*-icosanoyl-*N*-pantothenoylcysteamine (**10i**).

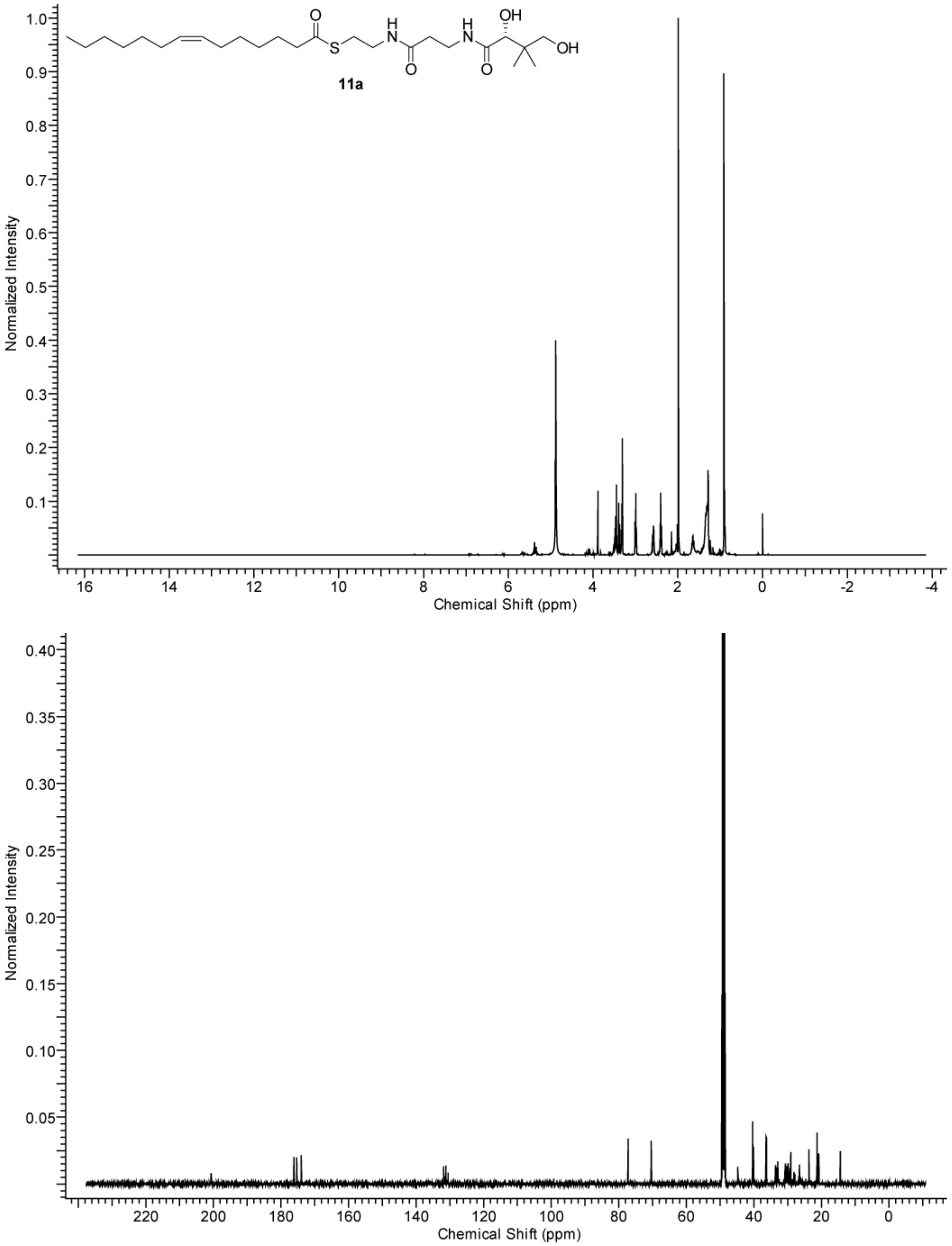

Figure S13: ^{1}H NMR and ^{13}C NMR spectrum of *S*-((*Z*)-7-tetradecenoyl)-*N*-pantothenoylcysteamine (**11a**).

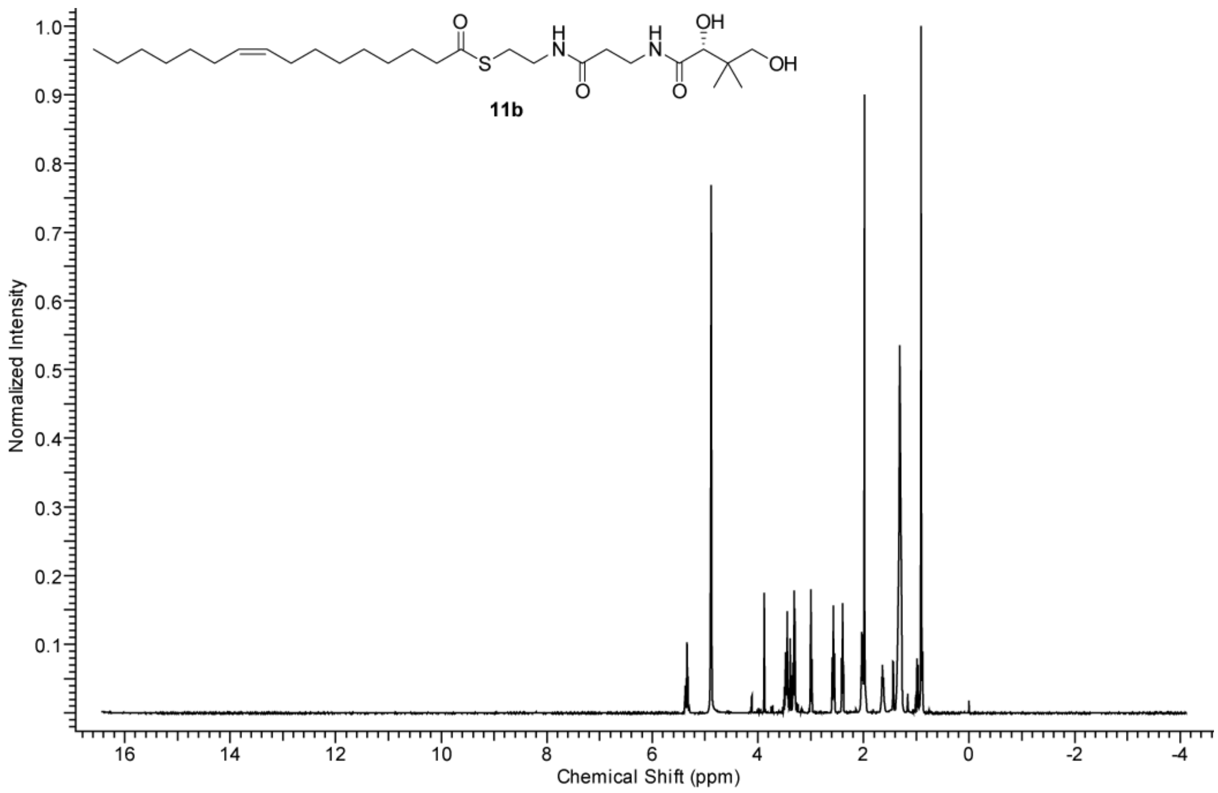

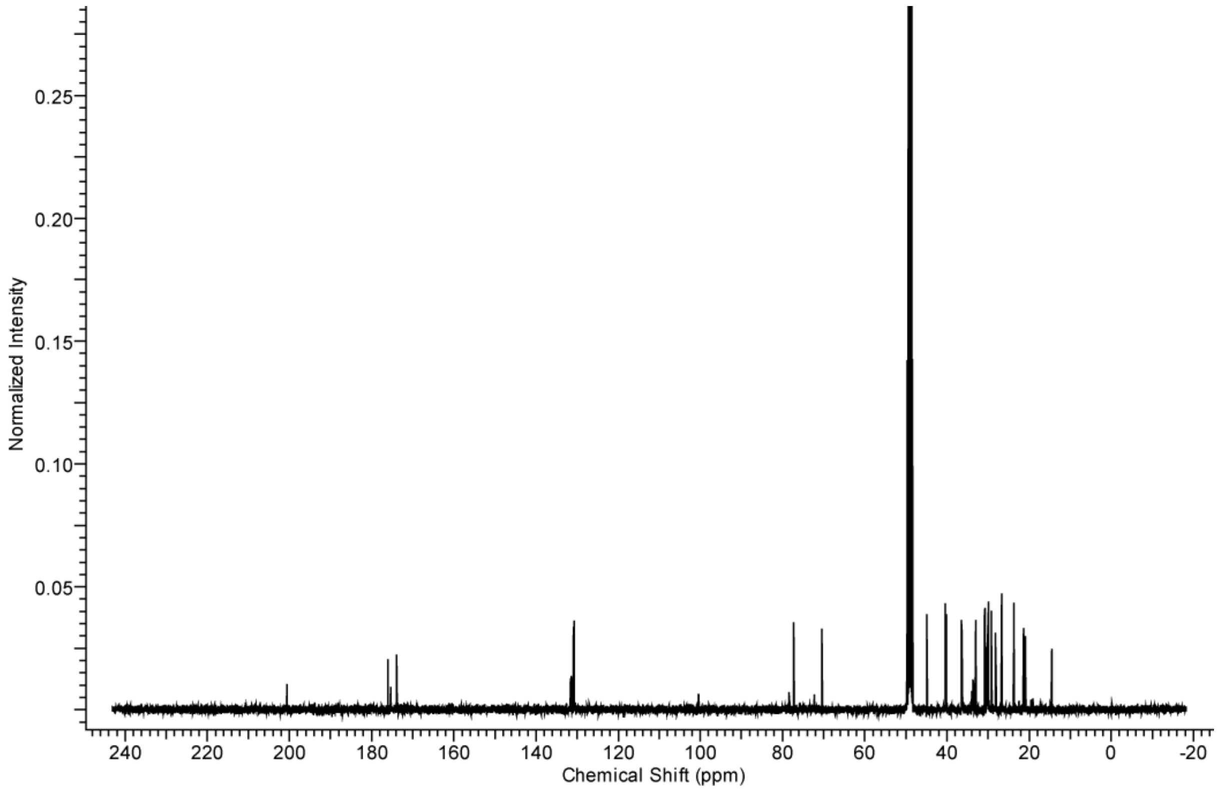

Figure S14: ^{1}H NMR and ^{13}C NMR spectrum of *S*-((*Z*)-9-hexadecenoyl)-*N*-pantothenoylcysteamine (**11b**).

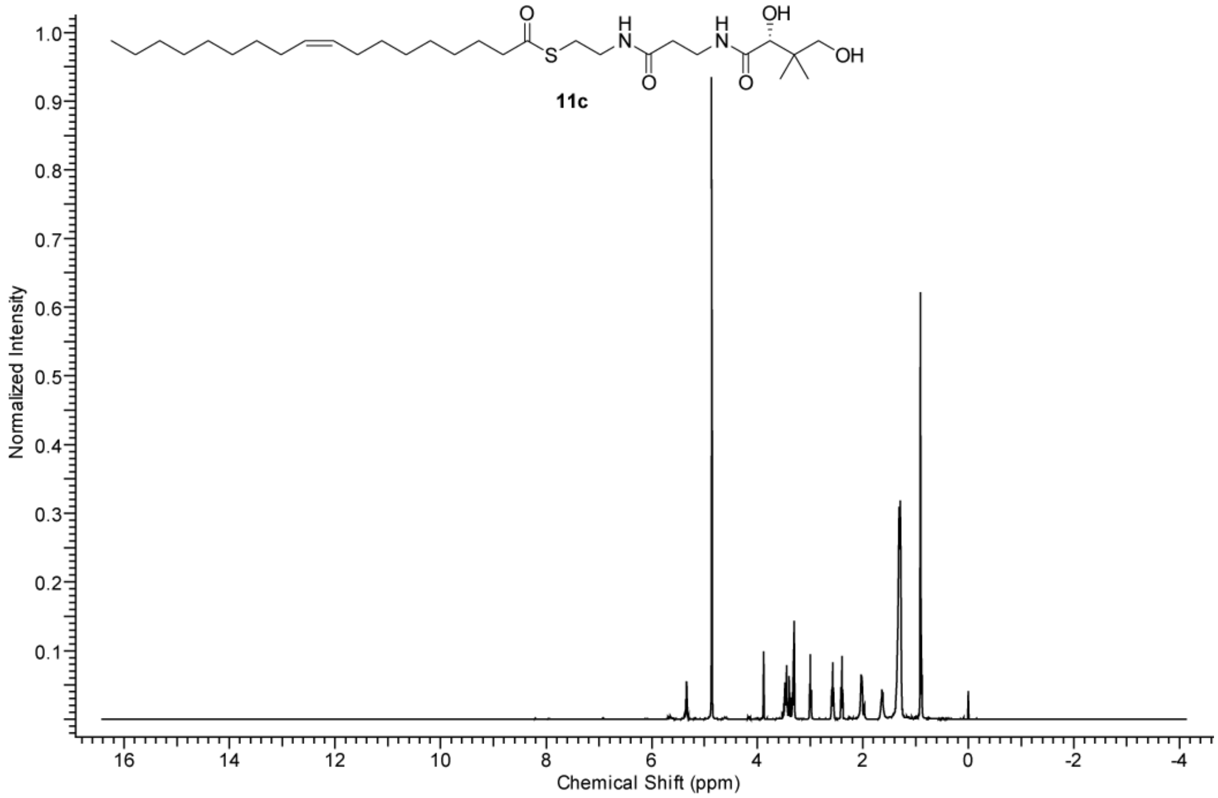

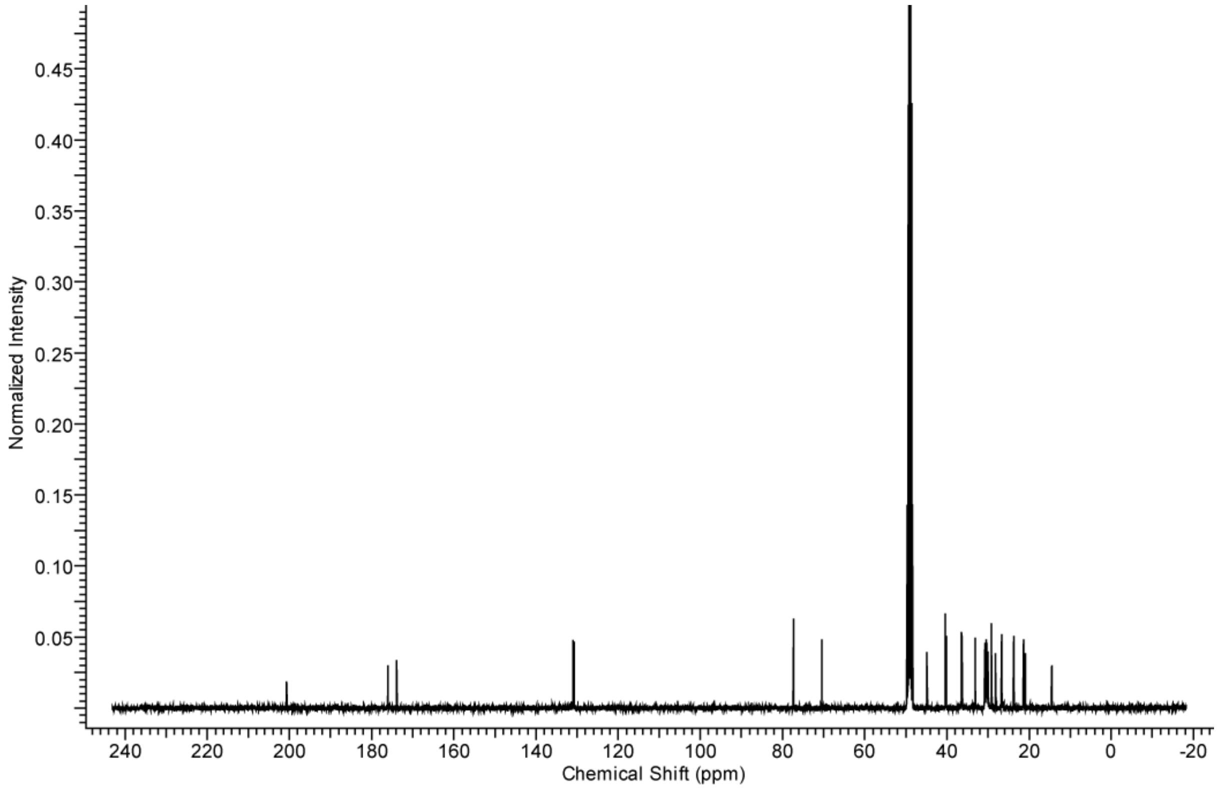

Figure S15: ^{1}H NMR and ^{13}C NMR spectrum of *S*-((*Z*)-9-octadecenoyl)-*N*-pantothenoylcysteamine (**11c**).

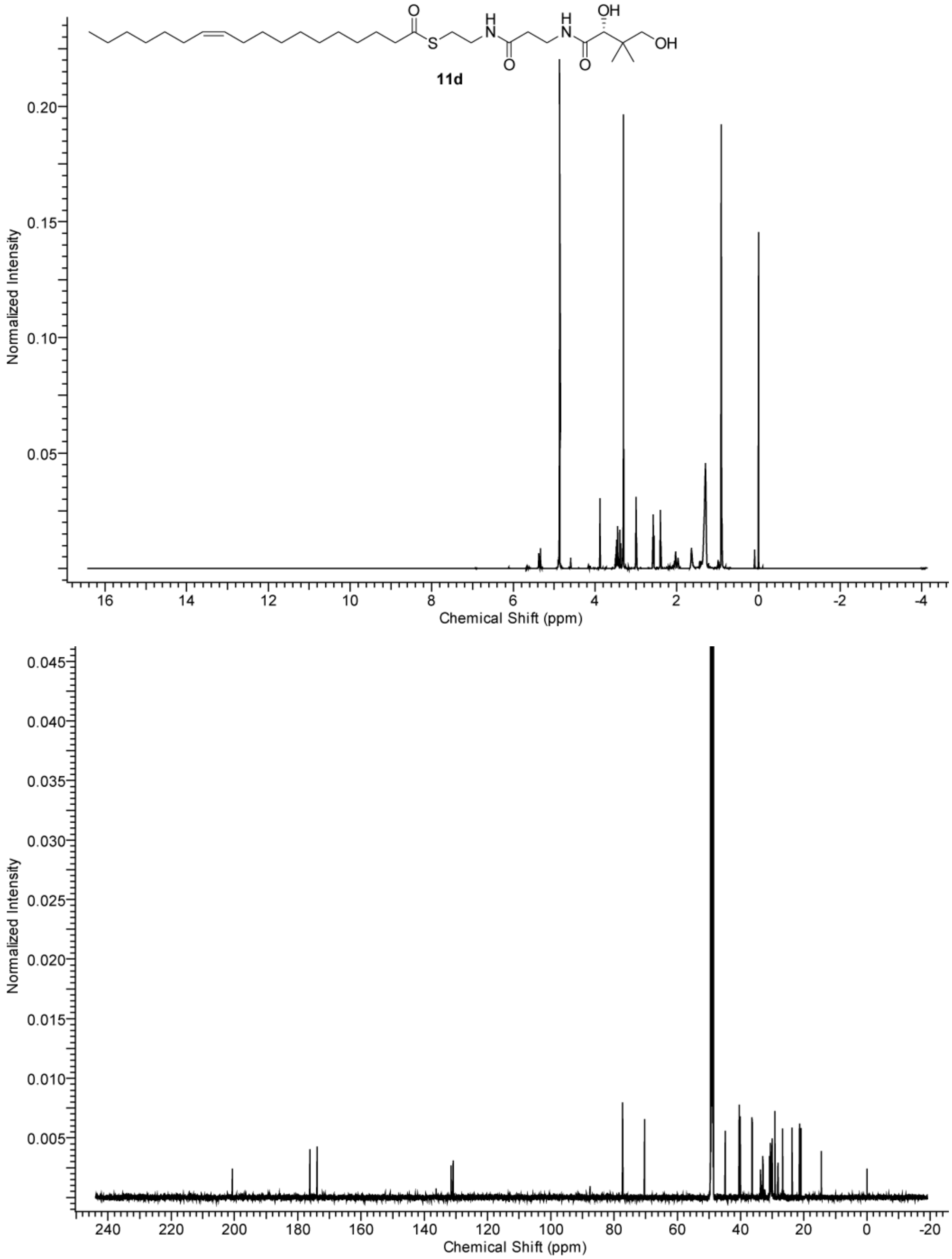

Figure S16: ^{1}H NMR and ^{13}C NMR spectrum of *S*-((*Z*)-11-octadecenoyl)-*N*-pantothenoylcysteamine (**11d**).

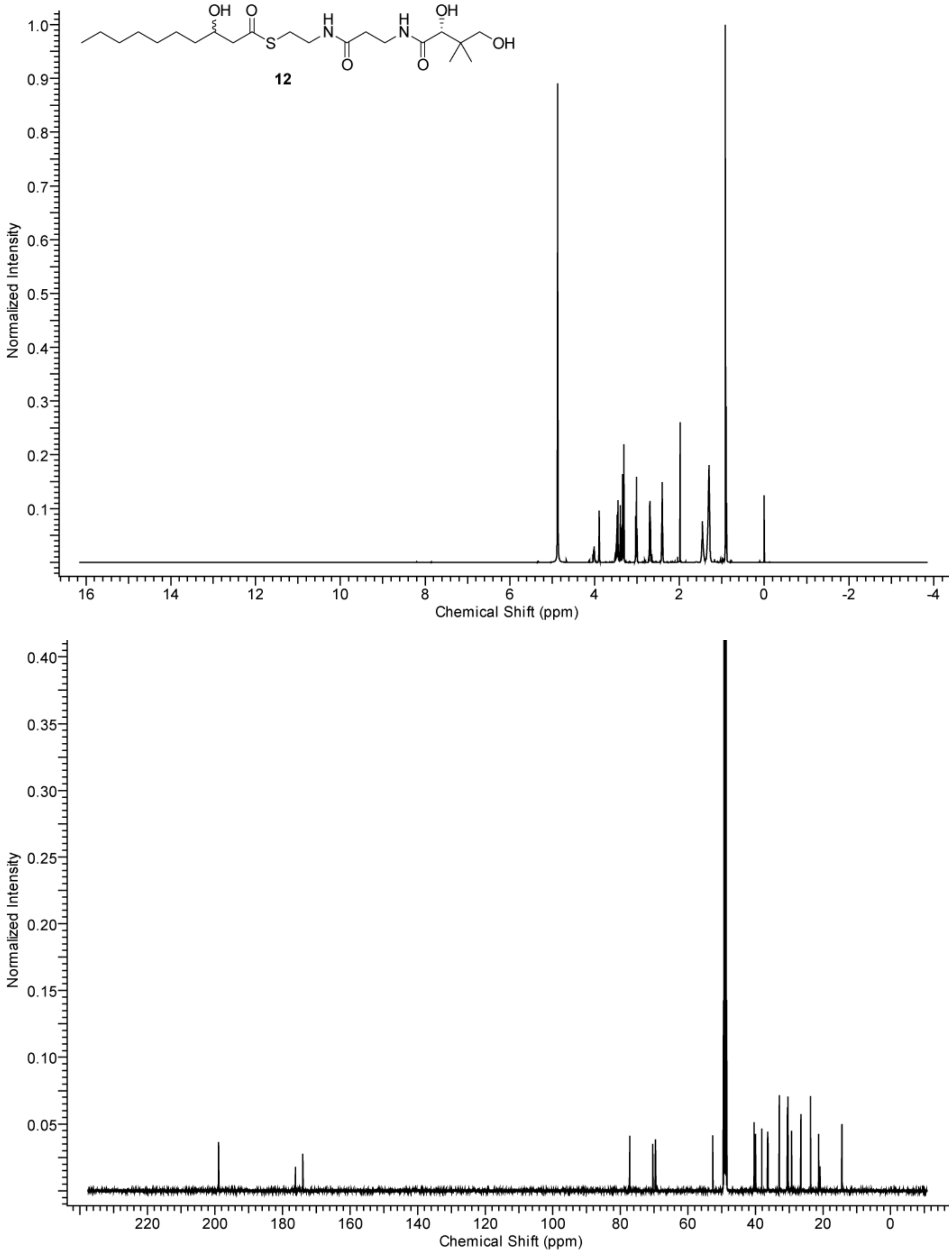

Figure S17: ^{1}H NMR and ^{13}C NMR spectrum of *S*-(3-hydroxydecanoyl)-*N*-pantothenoylcysteamine (**12**).

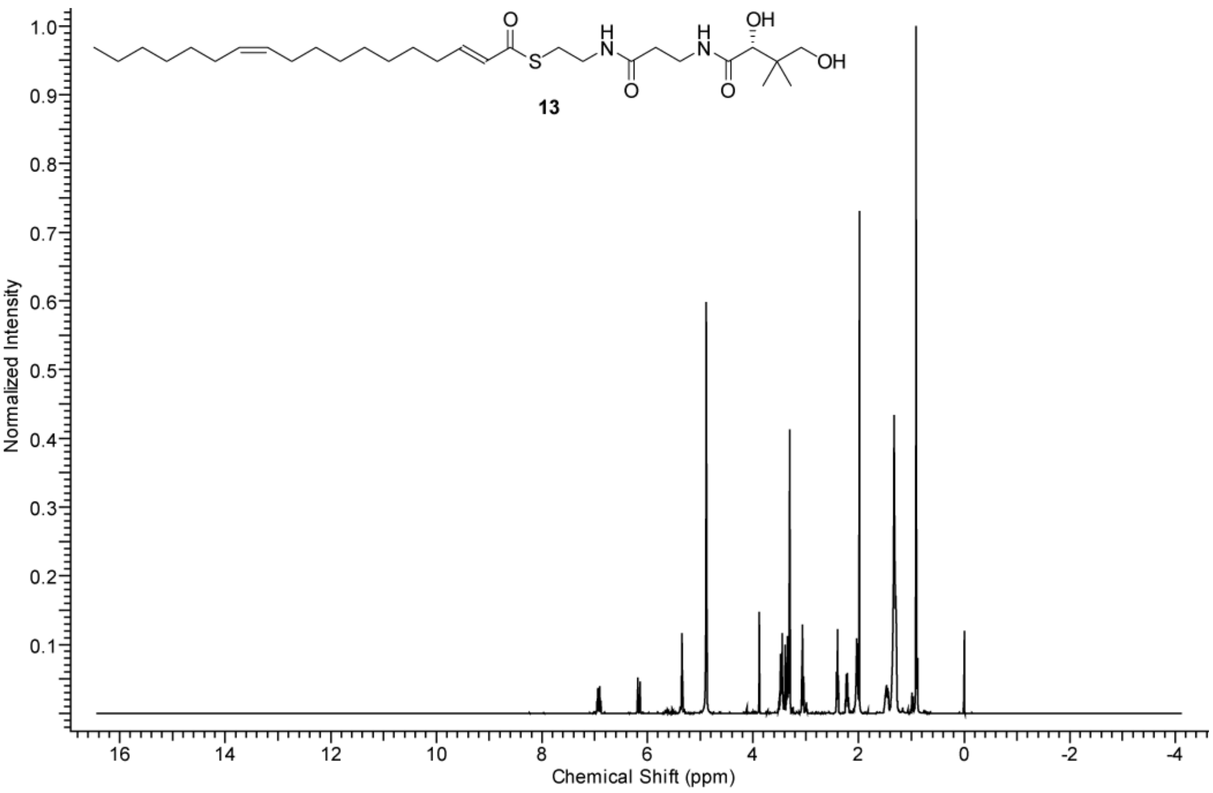

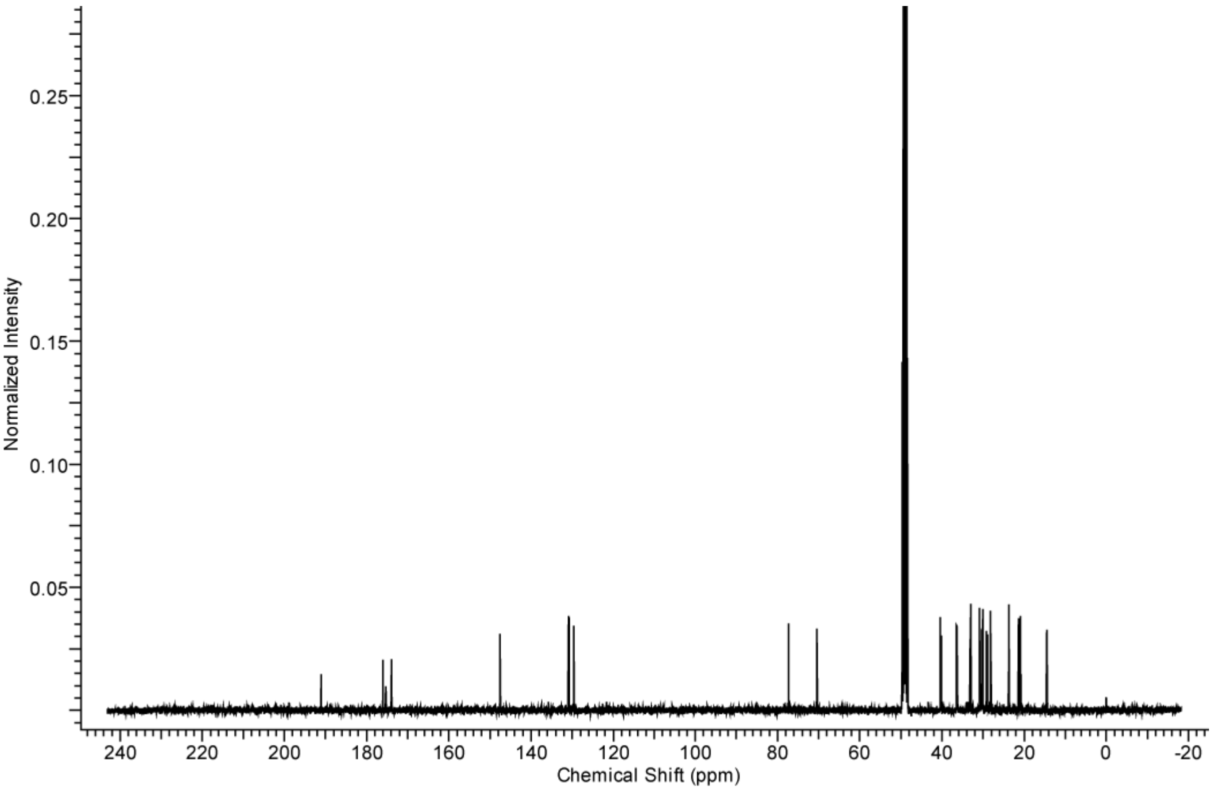

Figure S18: ^{1}H NMR and ^{13}C NMR spectrum of *S*-((2*E*,11*Z*)-2,11-octadecadienoyl)-*N*-pantothenoylcysteamine (**13**).
